# Control of criticality and computation in spiking neuromorphic networks with plasticity

**DOI:** 10.1038/s41467-020-16548-3

**Published:** 2020-06-05

**Authors:** Benjamin Cramer, David Stöckel, Markus Kreft, Michael Wibral, Johannes Schemmel, Karlheinz Meier, Viola Priesemann

**Affiliations:** 1 Kirchhoff-Institute for Physics, Heidelberg University, Im Neuenheimer Feld 227, 69120 Heidelberg, Germany; 2 Campus Institute for Dynamics of Biological Networks, Georg-August University, Hermann-Rein-Straße 3, 37075 Göttingen, Germany; 3 Max-Planck-Institute for Dynamics and Self-Organization, Am Faßberg 17, 37077 Göttingen, Germany; 4 Bernstein Center for Computational Neuroscience, Georg-August University, Am Faßberg 17, 37077 Göttingen, Germany; 5 Department of Physics, Georg-August University, Friedrich-Hund-Platz 1, 37077 Göttingen, Germany

**Keywords:** Dynamical systems, Electrical and electronic engineering, Computational science, Statistical physics, thermodynamics and nonlinear dynamics

## Abstract

The critical state is assumed to be optimal for any computation in recurrent neural networks, because criticality maximizes a number of abstract computational properties. We challenge this assumption by evaluating the performance of a spiking recurrent neural network on a set of tasks of varying complexity at - and away from critical network dynamics. To that end, we developed a plastic spiking network on a neuromorphic chip. We show that the distance to criticality can be easily adapted by changing the input strength, and then demonstrate a clear relation between criticality, task-performance and information-theoretic fingerprint. Whereas the information-theoretic measures all show that network capacity is maximal at criticality, only the complex tasks profit from criticality, whereas simple tasks suffer. Thereby, we challenge the general assumption that criticality would be beneficial for any task, and provide instead an understanding of how the collective network state should be tuned to task requirement.

## Abstract (web summary)

Designing efficient artificial networks able to quickly converge to optimal performance for a given task remains a challenge. Here, the authors demonstrate a relation between criticality, task-performance and information theoretic fingerprint in a spiking neuromorphic network with synaptic plasticity.

## Introduction

A central challenge in the design of an artificial network is to initialize it such that it quickly reaches optimal performance for a given task. For recurrent networks, the concept of criticality presents such a guiding design principle^1–7^. At a critical point, typically realized as a second-order phase transition between order and chaos or stability and instability, a number of basic processing properties are maximized, including sensitivity, dynamic range, correlation length, information transfer, and susceptibility^8–12^. Because all these basic properties are maximized, it is widely believed that criticality is optimal for task performance^1,2,4–7,9^.

Tuning a system precisely to a critical point can be challenging. Thus ideally, the system self-organizes to criticality autonomously via local-learning rules. This is indeed feasible in various manners by modifying the synaptic strength depending on the pre- and postsynaptic neurons’ activity only^6,9,13–19^. The locality of the learning rules is key for biological and artificial networks where global information (e.g., task-performance error or activity of distant neurons) may be unavailable or costly to distribute. Recently, it has been shown that specific local-learning rules can even be harnessed more flexibly: a theoretical study suggests that recurrent networks with local, homeostatic learning rules can be tuned toward and away from criticality by simply adjusting the input strength^17^. This would enable one to sweep the entire range of collective dynamics from subcritical to critical to bursty, and assess the respective task performance.

Complementary to tuning collective network properties such as the distance to criticality, local-learning also enables networks to learn specific patterns or sequences^20,21^. For example, spike-timing dependent plasticity (STDP) shapes the connectivity, depending only on the timing of the activity of the pre- and postsynaptic neuron. STDP is central for any sequence learning—a central ingredient in language and motor learning^20,21^. Such learning could strongly speed up convergence, and enables a preshaping of the artificial network—akin to the shaping of biological networks during development by spontaneous activity.

Given diverse learning rules and task requirements, it may be questioned whether criticality is always optimal for processing, or whether each task may profit from a different state, as hypothesized in ref. ^10^. One could speculate that, e.g., the long correlation time at criticality on the one hand enables long memory retrieval, but on the other hand could be unfavorable if a task requires only little memory. However, the precise relation between the collective state, and specific task requirements is unknown.

When testing networks, the observed network performance is expected to depend crucially on the choice of the task. How can one then characterize performance independently of a specific task, such as classification or sequence memory? A natural framework to characterize and quantify processing of any local circuit in a task-independent manner builds on information theory^22,23^: classical information theory enables us to quantify the transfer of information between neurons, the information about the past input, as well as the storage of information. The storage of information can be measured within the network or as read out from one neuron. In addition, most recently classical mutual information is being generalized to more than two variables within the framework of partial information decomposition (PID)^23–26^. PID quantifies the unique and redundant contribution of each source variable to a target, but most importantly also enables a rigorous quantification of synergistic computation, a key contributor for any information integration^23–25,27,28^. Thereby information theory is a key stepping stone when linking local computation within a network, with global task performance.

Simulations of recurrent networks with plasticity become very slow with increasing size, because every membrane voltage and every synaptic strength has to be updated. Here, neuromorphic chips promise an accelerated and scalable alternative to neural network simulations^29–35^. To achieve an efficient implementation, physical emulation of synapses and neurons in electrical circuitry are very promising^36^. In such “neuromorphic chips,” all neurons operate in parallel, and thus the speed of computation is largely independent of the system size, and is instead determined by the time constants of the underlying physical neuron and synapse models—such as in the brain. Realizing such an implementation technically remains challenging, especially when using spiking neurons and flexible synaptic plasticity. The BrainScaleS 2 prototype system combines physical models of neurons and synapses^37^ with a general purpose processor carrying out plasticity^38^. In this system, the analog elements provide a speedup, energy efficiency, and enable scaling to very large systems, whereas the general purpose processor enables to set the desired learning rules flexibly. Thus with this neuromorphic chip, we can run the long-term learning experiments—required to study the network self-organization—within very short compute-time.

In the following, we investigate the relation between criticality, task-performance and information-theoretic fingerprint. To that end, we show that a spiking neuromorphic network with synaptic plasticity can be tuned toward and away from criticality by adjusting the input strength. We show that criticality is beneficial for solving complex tasks, but not the simple ones—challenging the common notion that criticality in general is optimal for computation. Methods from classical information theory as well as the novel framework of PID show that our networks indeed enfold their maximum capacity in the vicinity of the critical point. Moreover, the lagged mutual information between the stimulus and the activity of neurons allows to establish a relation between criticality (as set by the input strength) and task performance. Thereby, we provide an understanding how basic computational properties shape task performance.

## Results

### Model overview

**Fig. 1:**
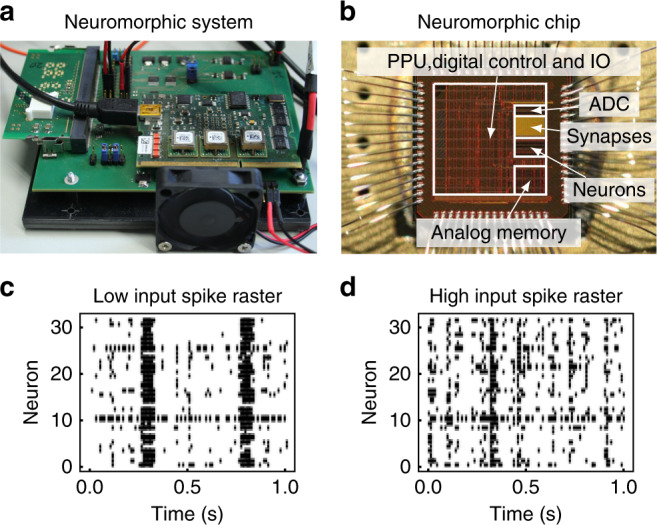
The degree of external input *K*_ext_ shapes the collective dynamics of the network. **a** The neural network is implemented on the prototype neuromorphic hardware system BrainScaleS 2. **b** This system features an analog-neural network core as well as an on-chip general purpose processor that allows for flexible plasticity implementation. **c** For low degree of input (*K*_ext_ = 0.25), strong recurrent connections develop, and the activity shows irregular bursts, resembling a critical state. **d** For high degrees of input (*K*_ext_ = 0.56), firing becomes more irregular and asynchronous.

We emulate networks of leaky integrate-and-fire (LIF) neurons on the mixed-signal neuromorphic prototype system BrainScaleS 2, which has *N* = 32 neurons (Fig. 1 a, b). Future versions of the BrainScaleS 2 chip will feature 512 neuron circuits with adaptive-exponential LIF dynamics and intercompartmental conductances. We use the term emulation in order to clearly distinguish between the physical implementation, where each observable has a measurable counterpart on the neuromorphic chip, and standard software simulations on conventional hardware. The system features an array of 32 × 32 current-based synapses, where 20% of the synapses are programmed to be inhibitory. Synaptic plasticity acts equally on all synapses and is composed of a positive drift and a negative anticausal STDP term. In conjunction both terms lead to homeostatic regulation and thus stable network activity of about 20 Hz per neuron (Supplementary Fig. 1a). Plasticity is executed by an on-chip general purpose processor alongside to the analog emulation of neurons and synapses. This allows for an uninterrupted and fast data acquisition. Even for the small prototype system, the advantages of neuromorphic computing in terms of speed and energy efficiency become important as depicted in ref. ^39^.

Neurons are potentially all-to-all connected, but *K*_ext_ out of the *N* synapses per neuron are used to inject external Poisson or pattern input. Effectively, *K*_ext_ quantifies the input strength with the extreme cases of *K*_ext_/*N* = 1 for a feed-forward network and *K*_ext_/*N* = 0 for a fully connected recurrent network, which is completely decoupled from the input. Depending on the degree of external input *K*_ext_, the network shows diverse dynamics (Fig. 1c, d). As expected^17^, *K*_ext_ shapes the collective dynamics of the network from synchronized for low *K*_ext_ to more asynchronous irregular for high *K*_ext_.

### Critical dynamics arise under low input *K*_ext_

**Fig. 2:**
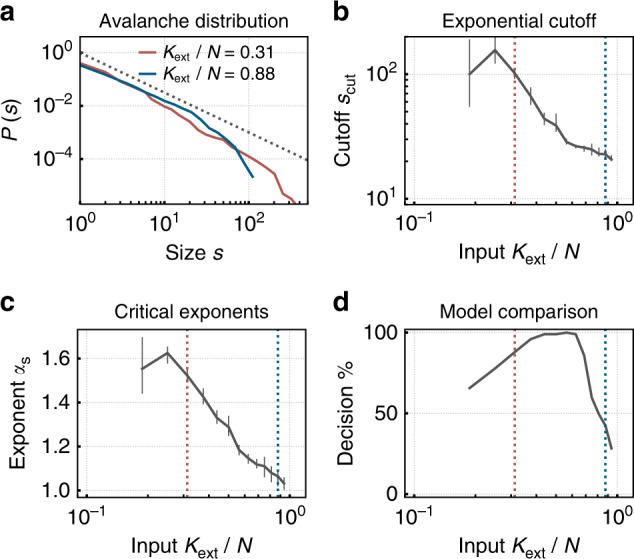
Under low degree of input *K*_ext_, the network self-organizes toward a critical state, and shows long-tailed avalanche distributions. **a** Distributions of avalanche sizes ${\mathcal{P}}(s)$ show power laws over two orders of magnitude for low *K*_ext_. Fitting a truncated power law, **b** the exponential cutoff *s*_cut_ peaks, and **c** critical exponents *α*_*s*_ approximate 1.5, as expected for critical branching processes. **d** A maximum-likelihood comparison decides for a power law compared with an exponential fit in the majority of cases. Dashed vertical lines indicate the set of *K*_ext_/*N* values that have been selected in **a**. In this and all following figures, the median over runs and (if acquired) trials is shown, and the errorbars show the 5–95% confidence intervals.

**Fig. 3:**
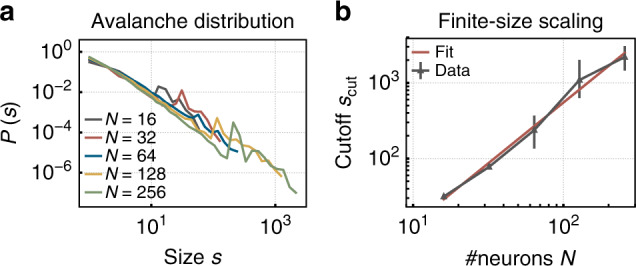
Finite-size scaling is assessed using a software implementation with varying system size *N*. **a** Exemplary avalanche size distributions ${\mathcal{P}}(s)$ follow a power law for any tested *N* (degree of input *K*_ext_/*N* = 1/4). **b** As expected for critical systems, the cutoff *s*_cut_ scales with the system size. The scaling exponent is 1.6 ± 0.2.

**Fig. 4:**
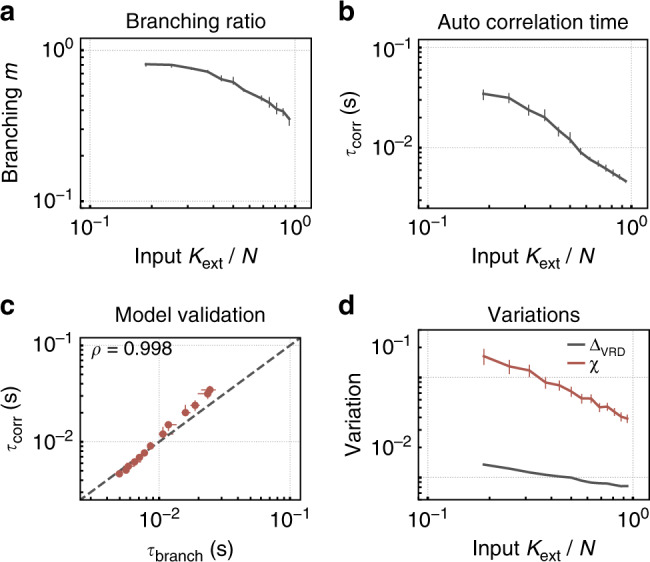
For low degree of input *K*_ext_, the network shows clear signatures of criticality beyond power laws. Only for low values of *K*_ext_, **a** the estimated branching ratio *m* tends toward unity, and **b** the estimated autocorrelation time *τ*_corr_ peaks. **c** The match of the *τ*_corr_, and the ${\tau }_{{\rm{branch}}} \sim -1/\mathrm{log}\,(m)$ as inferred from *m* supports the criticality hypothesis (correlation coefficient of *ρ* = 0.998, *p* < 10^−10^). **d** Trial-to-trial *Δ*_VRD_ variations as well as the susceptibility *χ* increase for low *K*_ext_.

The transition to burstiness for low *K*_ext_ suggests the emergence of critical dynamics, i.e., dynamics expected at a nonequilibrium second-order phase transition. Indeed, as detailed in the following, we find signatures of criticality in the classical avalanche distributions (Figs. 2 and 3) as well as in the branching ratio (Fig. 4a), the autocorrelation time (Fig. 4b), the susceptibility, and in trial-to-trial variations (Fig. 4d).

To test whether the network indeed approaches criticality, we assume the established framework of a branching process^8,40–42^. In branching processes, a spike at time *t* triggers on average *m* postsynaptic spikes at time *t* + 1, where *m* is called the branching parameter. For *m* = 1 the process is critical, and the dynamics give rise to large cascades of activity, called avalanches^8,43^. The size *s* of an avalanche is the total number of spikes in a cluster and is power law distributed at criticality. The binwidth for the estimation of the underlying distributions is set to the mean inter-event interval following common methods^44^. Our network shows power law distributed avalanche sizes *s* over two orders of magnitude for low *K*_ext_ (Fig. 2a). For almost any *K*_ext_, the distribution is better fitted by a power law than by an exponential distribution^45^ (Fig. 2d). However, only for low *K*_ext_ the exponent of the avalanche distribution is close to the expected one, *α*_*s*_ ≈ 1.5 (Fig. 2c), and the power law shows the largest cutoff *s*_cut_ (Fig. 2b). For low *K*_ext_, the networks tend to get unstable due to the limited number of neurons explaining the decline in *s*_cut_ (Fig. 2b) and in the maximum-likelihood comparison (Fig. 2d). Together, all the quantitative assessment of the avalanches indicate that a low degree of input *K*_ext_ produces critical-like behavior.

In a control experiment, we investigate finite-size scaling in software simulations, as the current physical system features only 32 neurons. Therefore, a network with the same topology, plasticity rules, and single-neuron dynamics (though without parameter noise and hardware constraints) is simulated for various system sizes *N*. The resulting avalanche distributions show power laws for any system size (Fig. 3a), and the cutoff *s*_cut_ scales with *N* as expected at criticality (Fig. 3b). The scaling exponent is 1.6 ± 0.2. Together, these numerical results confirm the hypothesis that for low degrees of input *K*_ext_, the small network that is emulated on the chip self-organizes as close to a critical state as possible.

The implementation on neuromorphic hardware promises fast emulation. Already for *N* = 32, the neuromorphic chip is about a factor of 100 faster than the Brian 2 simulation. To give numbers, a single plasticity experiment with a duration of 600 s biological time is simulated in 570 s on a single core of a Intel Xeon E5-2670 CPU in Brian 2, but emulated in only 6 s on the neuromorphic chip. Hence, a neuromorphic implementation is very promising especially for the future full size chip: When running such detailed networks as classical simulations, the computational overhead scales with ${\mathcal{O}}({N}^{2})$ due to the all-to-all connectivity and synaptic plasticity on conventional hardware. In contrast, for the neuromorphic system, the execution time is largely independent of the system size *N*, as long as the network can be implemented on the system.

The assumption that the critical state of the network corresponds to the universality class of critical branching processes is tested further by properly inferring the branching parameter *m* (Eq. (17)), the autocorrelation times, and the response to perturbations. First, the branching parameter *m* characterizes the spread of activity and is smaller (larger) than unity for subcritical (supercritical) processes. For our model, it is always in the subcritical regime, but tends toward unity for low *K*_ext_ (Fig. 4a). Second, the autocorrelation time *τ*_corr_ is expected to diverge at criticality as ${\tau }_{{\rm{branch}}}(m) \sim {\mathrm{lim}\,}_{m\to 1}(-1/\mathrm{log}\,(m))=\infty$^42^. Indeed, *τ*_corr_ as estimated directly from the autocorrelation of the population activity is maximal for low *K*_ext_ (Fig. 4b). Third, the estimates of *m* and *τ*_corr_ are in theory related via the analytical relation ${\tau }_{{\rm{branch}}}(m) \sim -1/\mathrm{log}\,(m)$. This relation holds very precisely in the model (Fig. 4c, correlation coefficient *ρ* = 0.998, *p* < 10^−10^). Fourth, toward criticality, the response to any perturbation increases. The impact of a small perturbation is quantified by a variant of the van-Rossum distance *Δ*_VRD_ (Eq. (15)). It peaks for low degrees of the external input *K*_ext_ (Fig. 4d). Last, one advantage of operating in the vicinity of a critical point is the ability to enhance stimulus differences by the system response. This is reflected in a divergence of the susceptibility at the critical point. The susceptibility *χ* (Eq. (16)), quantified here as the change in the population firing rate in response to a burst of *N*_pert_ = 6 additional spikes, is highest for low *K*_ext_ (Fig. 4d). Thus overall, the avalanche distributions as well as the dynamic properties of the network all indicate that it self-organizes to a critical point under low degree of input *K*_ext_.

### Network properties have to be tuned to task requirements

It is widely assumed that criticality optimizes task performance. However, we found that one has to phrase this statement more carefully. While certain abstract computational properties, such as the susceptibility, sensitivity, or memory time span are indeed maximal or even approaching infinity at a critical state, this is not necessary for task performance in general^5,11,42,46^. We find that it can even be detrimental. For every single task complexity, a different distance to criticality is optimal, as outlined in the following.

We study the performance of our recurrent neural network in the framework of reservoir computing: the performance of a recurrent neural network is quantified by the ability of linear readout neurons to separate different sequences^47–49^. To that end, it is often necessary to maintain information about past input for long time spans. To test performance, we specifically use a *n*-bit sum and a *n*-bit parity task and trained a readout on the activity of *N*_read_ = 16 randomly chosen neurons of the reservoir. For the two given tasks complexity increases with *n*: to solve the tasks, the network has to both *memorize* and *process* the input from the *n* past steps. As reservoirs close to a critical point have longer memory as quantified by the lagged mutual information (*I*_*τ*_, Fig. 7), one expects that particularly the memory intensive tasks profit from criticality (tasks with high *n* are better at low degrees of input *K*_ext_). In contrast, simple tasks (low *n*) might suffer from criticality because of the maintenance of memory about unnecessary input. Since the estimation of parity, in contrast to the sum, is fully nonlinear, their direct comparison allows to further dissect task complexity. Thus, depending on the task complexity, there should be an ideal *K*_ext_, leading to maximal performance.

**Fig. 5:**
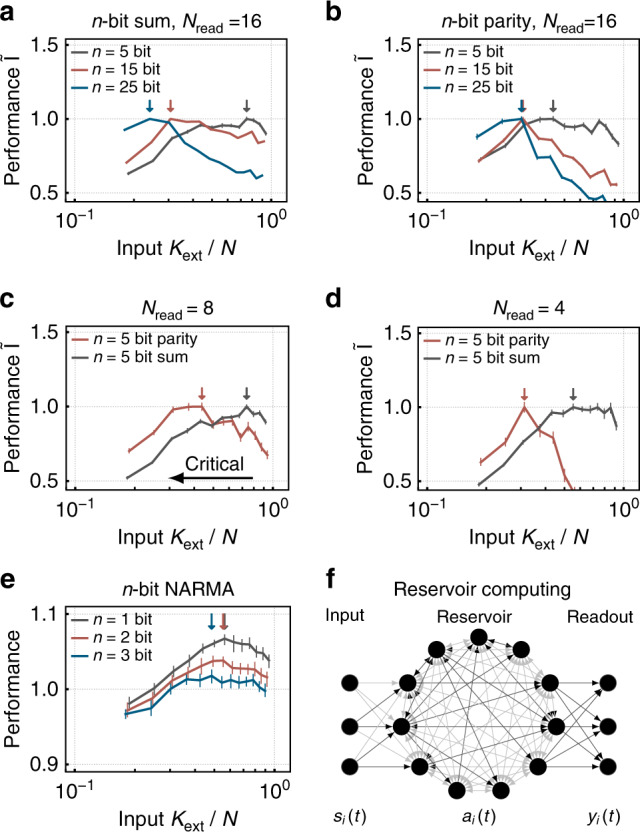
Computational challenging tasks profit from critical network dynamics (small *K*_ext_)—simple tasks do not. The network is used to solve (**a**) a *n*-bit sum and (**b**) a *n*-bit parity task by training a linear classifier on the activity of *N*_read_ = 16 neurons. Here, task complexity increases with *n*, the number of past inputs that need to be memorized and processed. For high *n*, task performance profits from criticality, whereas simple tasks suffer from criticality. Especially, the more complex, nonlinear parity tasks profits from criticality. Further, task complexity can be increased by restricting the classifier to **c** *N*_read_ = 8 and **d** *N*_read_ = 4. Again, the parity task increasingly profits from criticality with decreasing *N*_read_. The performance is quantified by the normalized mutual information $\tilde{{\rm{I}}}$ between the vote of the classifier and the parity or sum of the input. **e** Likewise, the peak performance moves toward criticality with increasing complexity in a NARMA task. The performance is quantified by the inverse NRMSE. Highest performance for a given task is highlighted by colored arrows. **f** Schematic reservoir computing setup.

For our network, we find indeed that maximal task performance depends on both, task complexity and distance to criticality: simple sum tasks (*n* = 5) are optimally solved away from criticality, whereas complex sum tasks (*n* = 25) profit from the long timescales arising at criticality (Fig. 5a). The nonlinear parity task profits even more from criticality: even for *n* = 5 networks closer to the critical point promote task performance (Fig. 5b). Hence, we are capable of adapting the networks computational properties to task complexity by fine-tuning the strength of the input.

Likewise, we investigated the ability of our networks to do combinations of operations by considering the classic nonlinear autoregressive moving average (NARMA) task^48^. The network’s peak performance again moves toward criticality with higher task complexity (Fig. 5e).

To further tune the difficulty of task, we reduced the number of neurons visible to the readout *N*_read_. We expect that in principle information about, e.g., parity could be available in a single neuron if the network is sufficiently close to criticality, because critical network dynamics are not only characterized by temporal, but also spatial correlations. The ability to condense information about extended stimuli in the activity of few neurons can be valuable. To quantify the effect of spatial correlations on computation, we trained linear classifiers on the activity of a subset of neurons for the 5-bit sum and the parity task. When lowering *N*_read_ from 8 to 4, only the nonlinear parity tasks increasingly profits from critical network dynamics (Fig. 5c, d). In contrast, the information necessary to solve the linear sum task seems to be globally available in the network response even for subcritical dynamics. The ability to locally read out global information from the network is of equal importance for both, large neuromorphic systems^29^ and living networks^50,51^.

### Adaptation to task by dynamic switching of input strengths

**Fig. 6:**
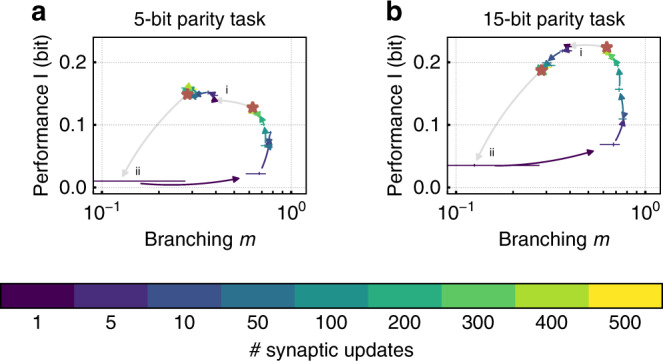
The network can be dynamically adapted by changing the degree of the input *K*_ext_. After convergence of synaptic weights *w*_*ij*_, *K*_ext_/*N* is (i) switched from critical (*K*_ext_ = 0.3) to subcritical (*K*_ex_ = 0.8) and (ii) vice versa. The branching ratio *m* and the performance I of the network on **a** the 5-bit and **b** the 15-bit parity task are evaluated after various numbers of synaptic updates. The network reaches the same performance and dynamics as when starting from *w*_*ij*_ = 0 ∀ *i*, *j* (marked by red stars). For both tasks, the transition from subcritical to critical dynamics requires more updates as expected. Moreover, optimal performance for (**a**) the 5-bit task is achieved under strong input (i), whereas (**b**) the 15-bit task requires low input (ii). The performance is quantified by the mutual information I between the parity of the input and the vote of a linear classifier.

We know from the previous experiments that for high *n*, the *n*-bit parity task is solved best at criticality, whereas for low *n*, the subcritical regime leads to best performance. In the following, we investigate how to transit between both states. To achieve this, we take the state of a critical network and switch the degree of the input *K*_ext_ to a subcritical configuration and vice versa. The performance is evaluated after various numbers of synaptic updates. This task switch generates the same working points as the previous emulations that start with synaptic weights *w*_*ij*_ = 0 ∀ *i*, *j* and have a long adaptation phase (red stars in Fig. 6).

A fast adaption to different input strengths is required to switch between tasks of different complexity. The transition from critical to subcritical is achieved after the application of about 50 synaptic updates corresponding to 50 s biological time, whereas going from subcritical to critical takes about 500 updates and therefore 500 s (Fig. 6). However, due to the speedup of the neuromorphic chip, the adaptation takes only about 0.5 s wall clock time and can even be lowered by decreasing the integration time over spike pairs in the synaptic update rule. As alternative strategies, one could switch between saved configurations, or run a hierarchy of networks with different working points in parallel^52^.

### Task-independent quantification of computational properties

**Fig. 7:**
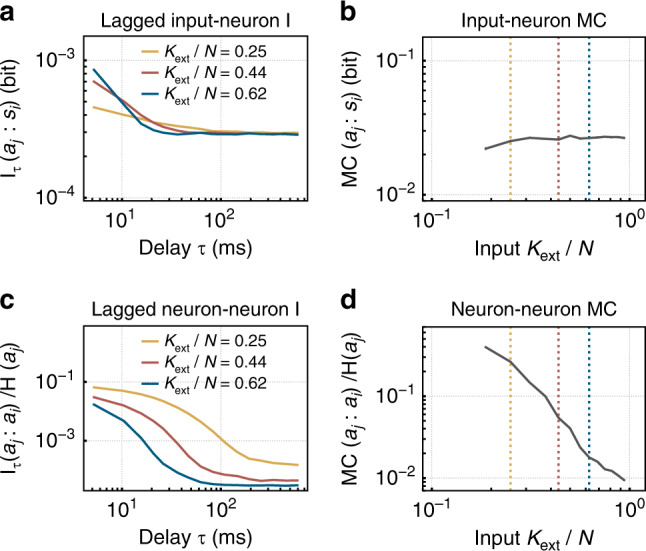
Long lasting memory accompanies critical network dynamics. **a** Memory about the input *s*_*i*_ as read out from neuron *a*_*j*_ after a time lag *τ* is quantified by the mutual information I_*τ*_(*a*_*j*_, *s*_*i*_). Here, high degrees of the external input *K*_ext_ are favorable for memory on short timescales, whereas low *K*_ext_ is favorable on larger timescales. **b** The memory capacity (MC) stays fairly constant, despite of a decreased coupling to the stimulus for low *K*_ext_. **c** The lagged I between the activity of pairs of neurons indicates increasing memory for decreasing *K*_ext_, also visible in the memory capacity (MC) (**d**). The selection of *K*_ext_/*N* in **a** and **c** is marked by dashed vertical lines in **b** and **d**.

While task performance is the standard benchmark for any model, such benchmark tasks have two disadvantages: in many biological systems, such as higher brain areas or in vitro preparations, such tasks cannot be applied. Even if tasks can be applied, the outcome will always depend on the chosen task. To quantify computational properties in a task-independent manner, information theory offers powerful tools^23^. Using the Poisson noise input, we find that the lagged mutual information I_*τ*_ between the input *s*_*i*_ and the activity of a neuron after a time lag *τ*, *a*_*j*_ predicts the performance on the parity task. Here, at high *K*_ext_ (away from criticality) information about the input is maximal for very short *τ*, but decays very quickly (Fig. 7a). This fast forgetting is important to irradiate past, task-irrelevant input that would interfere with novel, task-relevant input. At small *K*_ext_, the recurrence is stronger and input can be read out for much longer delays (20 ms vs. 60 ms). This active storage of information is required in a reservoir to solve any task that combines past and present input, and hence the more complex parity task also profits from it. However, the representation of input in every single neuron becomes less reliable (i.e., *I*_*τ*_ is smaller). A measure for the representation of the input in the network could be obtained by integrating I_*τ*_ over *τ*. Interestingly, this memory capacity (MC) stays fairly constant (Fig. 7b). Note that we only quantified the representation of the input in a single neuron, a measure very easily accessible in experiments; obviously the readout can draw on the distributed memory across all neurons, which jointly provide a much better readout.

The memory maintenance for task processing has to be realized mainly by activity propagating on the recurrent connections in the network. Therefore, it is often termed active information storage (AIS)^53^. The recurrent connections become stronger closer to criticality, and as a consequence we find that the lagged mutual information between pairs of neurons in the reservoir also increases (Fig. 7c). As a result the MC of the reservoir increases over almost two orders of magnitude when approximating criticality (lower *K*_ext_, Fig. 7d). This increase in internal MC carries the performance on the more complex parity tasks.

**Fig. 8:**
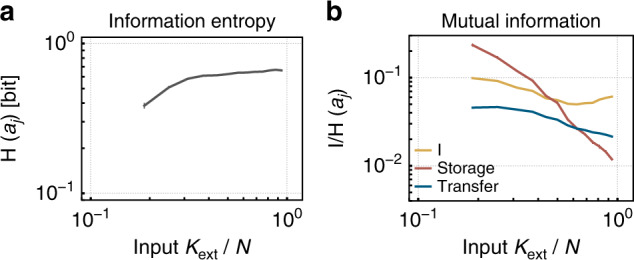
The information fingerprint changes with the degree of input *K*_ext_, thus with distance to criticality. **a** The entropy (H) of the spiking activity of a single neuron, *a*_*j*_ stays fairly constant, except for low *K*_ext_ as a consequence of decreasing firing rates. **b** The mutual information (I) between the activity of two units *a*_*i*_, *a*_*j*_ increases with lower *K*_ext_ (i.e., closer to critical). The network intrinsic memory also increases, indicated by the active information storage (AIS) ${\rm{I}}({a}_{j}:{{\bf{a}}}_{{\bf{j}}}^{-})$. Likewise, the information transfer within the network increases with lower *K*_ext_. Information transfer is measured as transfer entropy (TE) between pairs of neurons *a*_*j*_ and *a*_*i*_, ${\rm{I}}({a}_{j}:{{\bf{a}}}_{{\bf{i}}}^{-}| {{\bf{a}}}_{{\bf{j}}}^{-})$.

**Fig. 9:**
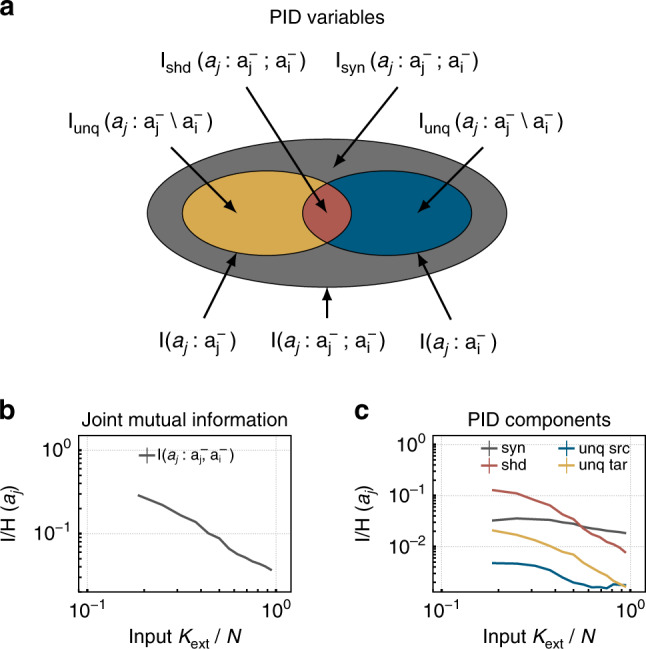
Partial information decomposition (PID) components increase toward criticality (i.e., with smaller input *K*_ext_). **a** The two input variables for PID correspond to the spiking histories ${{\bf{a}}}_{{\bf{i}}}^{-}$ and ${{\bf{a}}}_{{\bf{j}}}^{-}$ of two neurons, and the output variable to the present state *a*_*j*_. PID enables to quantify the unique contribution of each source to the firing of the target neuron (${{\rm{I}}}_{{\rm{unq}}}({a}_{j}:{{\bf{a}}}_{{\bf{j}}}^{-}\setminus {{\bf{a}}}_{{\bf{i}}}^{-})$ and ${{\rm{I}}}_{{\rm{unq}}}({a}_{j}:{{\bf{a}}}_{{\bf{i}}}^{-}\setminus {{\bf{a}}}_{{\bf{j}}}^{-})$), as well as the shared (also called redundant, ${{\rm{I}}}_{{\rm{shd}}}({a}_{j}:{{\bf{a}}}_{{\bf{j}}}^{-};{{\bf{a}}}_{{\bf{i}}}^{-})$), and synergistic contributions (${{\rm{I}}}_{{\rm{syn}}}({a}_{j}:{{\bf{a}}}_{{\bf{j}}}^{-},{{\bf{a}}}_{{\bf{i}}}^{-})$). **b** The joint mutual information (I) increases with decreasing *K*_ext_. **c** All PID components increase with approaching criticality. Interestingly, the synergistic and shared contributions are always much larger than the unique contributions (note the logarithmic axis). This highlights the collective nature of processing in recurrent neural networks.

When assessing computational capacities, information theory enables us to quantify not only the entropy (H) and mutual information (I) between units, but also to disentangle transfer and storage of information, as well as unique, redundant, and synergistic contributions of different source neurons^23–25,27,54^. We find that all these quantities increase with approaching criticality (smaller *K*_ext_, Figs. 8b and 9c). This indicates that the overall computational capacity of the model increases, as predicted for the vicinity of the critical state^1,2,7,11^.

In more detail, the AIS of a neuron, as well as the I and the transfer entropy (TE) between pairs of neurons increase with lower *K*_ext_ (Fig. 8b). In our analysis, these increases reflect memory that is realized as activity propagation on the network, and not storage within a single neuron, because the binsize used for analysis is larger than the refractory period *τ*_ref_, synaptic *τ*_syn_, and membrane-timescales *τ*_*m*_. Information theory here enables us to show that active transfer and storage of information within the network strongly increases toward criticality. A similar increase in I, AIS, and TE has been observed for the Ising model and reservoirs at criticality^1,11^, and hence supports the notion that criticality maximizes information processing capacity. Note however, that this maximal capacity is typically not necessary; as shown here, it can even be unfavorable when solving simple tasks.

Very recently, it has become possible to dissect further the contributions of different neurons to processing, using PID^24^. PID enables us to disentangle for a target neuron *a*_*i*_, how much unique information it obtains from its own past activity ${{\bf{a}}}_{{\bf{i}}}^{-}$, or the past activity of a second neuron ${{\bf{a}}}_{{\bf{j}}}^{-}$; and how much information is redundant or even synergistic from the two (Fig. 9a). Synergistic information is that part of information that can only be computed if both input variables are known, whereas redundant information can be obtained from one or the other.

All the PID components increase when approaching the critical point (low *K*_ext_, Fig. 9c). Quantitatively, the redundant and the synergistic information are always stronger than the unique ones that are about ten times less. The shared information dominates closer to criticality, mirroring the increased network synchrony and redundancy between neurons. Further, the synergistic contribution, i.e., the contributions that rely on the past of both neurons slightly increases, and is indeed the largest contribution for high *K*_ext_. This reflects that typically the joint activity of both neurons is required to activate a LIF neuron. Interestingly, the strong increase in shared information (i.e., redundancy) does not seem to impede the performance at criticality (small *K*_ext_). However, for even higher synchrony, as expected beyond this critical transition, the shared information might increase too much and thereby decrease performance.

## Discussion

In this study, we used a neuromorphic chip to emulate a network, subject to plasticity, and showed a clear relation between criticality, task-performance and information-theoretic fingerprint. Most interestingly, simple tasks do not profit from criticality while complex ones do, showing that every task requires its own network state.

The state and hence computational properties can readily be tuned by changing the input strength, and thus a critical state can be reached without any parameter fine-tuning within the network. This robust mechanism to adapt a network to task requirements is highly promising, especially for large networks where many parameters have to be tuned and in analog neuromorphic devices that are subject to noise in parameters and dynamics.

It has been generally suggested that criticality optimizes task performance^1,9^. We show that this statement has to be specified: indeed, criticality maximizes a number of properties, such as the autocorrelation time (Fig. 4b), the susceptibility (Fig. 4d), as well as information-theoretic measures (Figs. 7–9). However, this maximization is apparently not at all necessary, potentially even detrimental, when dealing with simple tasks. For our simple task, high network capacity results in maintenance of task-irrelevant information, and thereby harms performance. This is underlined by our results that clearly show that all abstract computational properties are maximized at criticality, but only the complex tasks profit from criticality. Hence, every task needs its own state and therefore a specific distance to critical dynamics.

The input strength could not only be controlled by changing *K*_ext_, the number of synapses of a neuron that were coupled to the input. An equally valid choice is a change of external input rate to each neuron. In fact, we showed that changing the input rate has the same effects on the relation between criticality (Supplementary Figs. 1–3), task performance (Supplementary Fig. 4), and information measures (Supplementary Figs. 5 and 6) as changing *K*_ext_. Moreover, in this framework the lowest input rates even allow to cross the critical point (Supplementary Figs. 3 and 4). Thus for both control mechanisms or a combination, there exists an optimal input strength, where the homeostatic mechanisms bring the network closest to critical. This optimal input strength has been derived analytically for a mean-field network by Zierenberg et al.^17^, and could potentially be used to predict the optimal input strength for other networks and tasks as well.

Not only the input strength, but also the strength of inhibition can act as a control parameter. Inhibition plays a role in shaping collective dynamics and is known to generate oscillations^55,56^. For a specific ratio of excitation and inhibition, criticality has been observed in neural networks^18,19,57,58^. Likewise, our networks has 20% inhibitory neurons. However, inhibition would not be necessary for criticality^13,42^. Nevertheless, the existence of more than one control parameters (degree of input, input rate, and inhibition) allows for flexible adjustment even in cases where only one of them could be freely set without perturbing input coding.

Plasticity plays a central role in self-organization of network dynamics and computational properties. In our model, the plasticity, neuron and synapse dynamics feature quite some level of biological detail (Table 1), and thus results could potentially depend on them. All synaptic weights are determined by the synaptic plasticity. Here, we showed results for homeostasis and STDP that implement the negative (anticausal) arm only. When implementing the positive (causal) arm of STDP in addition, the network destabilized, despite counteracting homeostasis. This is a well known problem^59^. Our implementation is still similar to full STDP, because anticausal correlations are weakened and the causal ones are indirectly strengthened by homeostasis. With its similarity to STDP and its inherent stability, our reduced implementation may be useful for future studies.

**Table 1:** Overview of the model parameters.

Parameter	Symbol	Value
Threshold potential	*u*_thresh_	(554 ± 21) mV
Leak potential	*u*_leak_	(384 ± 79) mV
Reset potential	*u*_reset_	(319 ± 18) mV
Membrane capacitance	*C*_*m*_	(2.38 ± 0.02) nF
Membrane time constant	*τ*_mem_	(1.6 ± 1.0) ms
Refractory period	*τ*_ref_	(4.9 ± 0.5) ms
Synaptic time constant	${\tau }_{{\rm{syn}}}^{{\rm{exc}}}$	(3.7 ± 0.5) ms
	${\tau }_{{\rm{syn}}}^{{\rm{inh}}}$	(2.8 ± 0.3) ms
Synaptic delay	*d*_syn_	(1.9 ± 0.1) ms
Weight scaling	*γ*	(8.96 ± 0.13) μA
Inhibitory synapses per neuron	*N*_inh_	6
Neurons	*N*	32
Degree of input	*K*_ext_	6–32
Input rate	*ν*	29 Hz
STDP time constant	*τ*_STDP_	(6.8 ± 1.2) ms
STDP amplitude	*η*	0.071 ± 0.023
Correlation scaling	*λ*_stdp_	11/128
Drift parameter	*λ*_drift_	1/512
Range of random variable	*n*_amp_	15/16
Bias of random variable	〈*n*〉	3/16
Burn-in experiment duration	*T*^burnin^	625 s
Static experiment duration	${T}^{\exp }$	104 s
Static trial experiment duration	*T*^static^	1 s
Training experiment duration	*T*^train^	104 s
Testing experiment duration	*T*^test^	21 s
Perturbation experiment duration	*T*^pert^	2 s
Perturbation time	*t*_pert_	1 s
Initial weight	${w}_{ij}^{{\rm{init}}}$	0 μA
Plasticity update period	*T*	1 ms
Embedding dimension	*l*	4
Delay steps	*N*_*τ*_	100

All time constants are given in biological time. Spike-timing dependent plasticity (STDP) amplitudes as well as time constants were measured using 20 spike pairs. The errors indicate the standard deviation.

The characterization of the network in a task-dependent as well as in a task-independent manner is essential for understanding the impact of criticality on computation. The computational properties in the vicinity of a critical point have been investigated by the classical measures AIS, I, and TE alone^5,60^, or by PID alone^27,61^. In this paper, we indeed showed that criticality maximizes capacity, but this does not necessarily translate to maximal task performance. Moreover, the lagged I between the stimulus and the activity of neurons allows to estimate memory timescales required to solve our tasks. This enables us to understand how task complexity and the information-theoretic fingerprint are related. Such understanding is the basis for well-founded design decisions of future artificial architectures.

The presented framework is particularly useful for analog neuromorphic devices as analog components have inherent parameter noise as well as thermal noise, which potentially destabilize the network. Here, the synaptic plasticity plays a key role in equalizing out particularly the parameter noise, as also demonstrated for short-term plasticity^62^, and thus makes knowledge about parameter variations, as well as specific calibration to some extend unnecessary.

Despite the small system size (*N* = 32 neurons only), the network not only showed signatures of criticality, but also developed quite complex computational capabilities, reflected in both, the task performance and the abstract information-theoretic quantities. We expect that a scale-up of the system size would open even richer possibilities. Such a scale-up would not even require fine-tuning of parameters, as the network self-tunes owing to the local-learning rules. As soon as larger chips are available, we expect that the abilities of neuromorphic hardware could be exhausted in terms of speed and energy efficiency allowing for long, large-scale, and powerful emulations.

Overall, we found a clear relation between criticality, task-performance and information-theoretic fingerprint. Our result contradicts the widespread statement that criticality is optimal for information processing in general: while the distance to criticality clearly impacts performance on the reservoir task, we showed that only the complex tasks profit from criticality; for simple ones, criticality is detrimental. Mechanistically, the optimal working point for each task can be set very easily under homeostasis by adapting the mean input strength. This shows how critical phenomena can be harnessed in the design and optimization of artificial networks, and may explain why biological neural networks operate not necessarily at criticality, but in the dynamically rich vicinity of a critical point, where they can tune their computational properties to task requirements^10,63^.

## Methods

We start with a description of the implemented network model, followed by a summary of the analysis techniques. All parameters are listed in Table 1 and all variables in the Supplementary Tables 1 and 2.

### Model

The results shown in this article are acquired on the mixed-signal neuromorphic hardware system described in ref. ^38^ (Fig. 1b). In the following a brief overview of the model, which is approximated by the physical implementation on the hardware, and the programmed plasticity rule is given. Since the neuromorphic hardware system comprises analog electric circuits, transistor mismatch causes parameter fluctuations, which can be compensated by calibration^37^. Here, no explicit calibration on the basis of single neurons and synapses is applied. Instead, only parameters common to all neurons/synapses are set such that all parts behave according to the listed equations, especially that all parts are sensible to input but silent in the absence of input. This choice leads to uncertainties in the model parameters as reported in Table 1.

Neurons: Implemented in analog circuitry, the neurons approximate current-based LIF neurons. The membrane potential *u*_*j*_ of the *j*-th neuron obeys

1 $${\tau }_{{\rm{mem}}}\frac{{\rm{d}}{u}_{j}}{\rm{d}t}=-\left[{u}_{j}(t)-{u}_{{\rm{leak}}}\right]+\frac{{I}_{j}(t)}{{g}_{{\rm{leak}}}}\ ,$$

with the membrane time constant *τ*_mem_, the leak conductance *g*_leak_ = *C*_*m*_/*τ*_mem_, the leak potential *u*_leak,_ and the input current *I*_*j*_(*t*). The *k*-th firing time of neuron *j*, ${t}_{j}^{k}$, is defined by a threshold criterion

2 $${t}_{j}^{k}:{u}_{j}({t}_{j}^{k})\ge {u}_{{\rm{thresh}}}\ .$$

Immediately after ${t}_{j}^{k}$, the membrane potential is clamped to the reset potential *u*_*j*_(*t*) = *u*_reset_ for $t\in \left({t}_{j}^{k},{t}_{j}^{k}+{\tau }_{{\rm{ref}}}\right]$, with the refractory period *τ*_ref_. The neuromorphic hardware system comprises *N* = 32 neurons, operating in continuous time due to the analog implementation.

Synapses: Like the membrane dynamics, the synapses are implemented in electrical circuits. Each neuron features *N* = 32 presynaptic partners (in-degree is 32). The synaptic input currents onto the *j*-th neuron enter the neuronal dynamics in Eq. (1) as the sum of the input currents of all presynaptic partners *i*, ${I}_{j}(t)=\mathop{\sum }\nolimits_{i = 1}^{N}{I}_{ij}(t)$, where *I*_*ij*_(*t*) is given by

3 $${\tau }_{{\rm{syn}}}^{{\rm{exc}}}\frac{{\rm{d}}{I}_{ij}(t)}{{\rm{d}}t}=-{I}_{ij}(t)+{I}_{ij}^{{\rm{ext}}}(t)+{I}_{ij}^{{\rm{rec}}}(t)\ ,$$

4 $${\tau }_{{\rm{syn}}}^{{\rm{inh}}}\frac{{\rm{d}}{I}_{ij}(t)}{{\rm{d}}t}=-{I}_{ij}(t)-{I}_{ij}^{{\rm{ext}}}(t)-{I}_{ij}^{{\rm{rec}}}(t)\ ,$$

with the excitatory and the inhibitory synaptic time constants ${\tau }_{{\rm{syn}}}^{{\rm{exc}}}$ and ${\tau }_{{\rm{syn}}}^{{\rm{inh}}}$. *N*_inh_ synapses of every neuron *j* are randomly selected to be inhibitory. The external synaptic current ${I}_{ij}^{{\rm{ext}}}(t)$ depends on the *l*-th spike time of an external stimulus *i*, ${s}_{i}^{l}$, whereas the recurrent synaptic current ${I}_{ij}^{{\rm{rec}}}(t)$ depends on the *k*-th spike time of neuron *i*, ${t}_{i}^{k}$, each of which transmitted to neuron *j*

5 $${I}_{ij}^{{\rm{ext}}}(t)=\sum _{l}\gamma \cdot {w}_{ij}^{{\rm{ext}}}\cdot \delta \left(t-{s}_{i}^{l}-{d}_{{\rm{syn}}}\right)\ ,$$

6 $${I}_{ij}^{{\rm{rec}}}(t)=\sum _{k}\gamma \cdot {w}_{ij}^{{\rm{rec}}}\cdot \delta \left(t-{t}_{i}^{k}-{d}_{{\rm{syn}}}\right)\ ,$$

with the synaptic delay *d*_syn_ and the weight conversion factor *γ*. The synaptic weight from an external spike source *i* to neuron *j* is denoted by ${w}_{ij}^{{\rm{ext}}}$, and ${w}_{ij}^{{\rm{rec}}}$ is the synaptic weight from neuron *i* to neuron *j*. Every synapse either transmits external events ${s}_{i}^{l}$ or recurrent spikes ${t}_{i}^{k}$, i.e., if ${w}_{ij}^{{\rm{stim}}}\ge 0$ then ${w}_{ij}^{{\rm{rec}}}=0$ and vice versa.

Network: The LIF neurons are potentially connected in an all-to-all fashion. A randomly selected set of *K*_ext_ synapses of every neuron is chosen to be connected to the spike sources. As every synapse could either transmit recurrent or input spikes, the *K*_ext_ synapses do not transmit recurrent spikes.

Plasticity: In the network, all synapses are plastic, the recurrent and the ones linked to the external input. Therefore, we skip the superscript of the synaptic weight and drop the distinction of ${t}_{i}^{k}$ and ${s}_{i}^{k}$ in the following description. Weights are subject to three contributions: a weight drift controlled by the parameter *λ*_drift_, a correlation sensitive part controlled by *λ*_stdp_, and positively biased noise contributions. This is very similar to STDP, however with specific depression, but unspecific potentiation. A specialized processor on the neuromorphic chip is programmed to update synaptic weights to *w*_*ij*_(*t* + *T*) = *w*_*ij*_(*t*) + *Δw*_*ij*_ according to

7 $$\Delta {w}_{ij}={\underbrace{-{\lambda}_{{\rm{stdp}}}f\left({t}_{i}^{k},{t}_{j}^{l},t\right)}_{{{\text{specific}} \, {\text{depression}}}}}-{\underbrace{{\lambda}_{{\rm{drift}}}{w}_{ij}}_{\text{decay}}}+{\underbrace{{n}_{ij}(t)}_{{\text{unspecific}} \, {\text{potentiation}}}}.$$

The STDP-kernel function *f* depends on the pre- and postsynaptic spike times in the time interval [*t* − *T*, *t*)

8 $$f\left({t}_{i}^{k},{t}_{j}^{l},t\right)=\sum _{\begin{array}{c}{t}_{i}^{k},{t}_{j}^{l}\end{array}}{\eta }_{{\rm{stdp}}}\exp \left(\frac{{t}_{j}^{l}-{t}_{i}^{k}}{{\tau }_{{\rm{stdp}}}}\right)\ ,$$

with ${t}_{i}^{k}\, > \, {t}_{j}^{l}$, and ${t}_{i}^{k},{t}_{j}^{l}\in [t-T,t)$, and only nearest-neighbor spike times are considered in the sum. *η*_stdp_ and *τ*_stdp_ denote the amplitude and the time constant of the STDP-kernel. The term *n*_*ij*_(*t*) adds a uniformly distributed, biased random variable

9 $${n}_{ij} \sim {\rm{unif}}\left(-{n}_{{\rm{amp}}},{n}_{{\rm{amp}}}\right)+\langle n\rangle \ ,$$

where *n*_amp_ specifies the range, while 〈*n*〉 is the bias of the random numbers.

The parameters *λ*_stdp_ and *λ*_drift_ are chosen such that the average combined force of the drift and the stochastic term is positive. Thus, only the negative arm of STDP is implemented.

Initialization: The synaptic weights are initialized to *w*_*ij*_ = 0 μA. Afterward, the network is stimulated by *N* Poisson-distributed spike trains of rate *ν* by the *K*_ext_ synapses of every neuron. By applying Eq. (7) for the total duration *T*^burnin^ weights *w*_*ij*_ ≠ 0 μA develop. For every *K*_ext_, the network is run 100 times, each with a different random seed. If not stated otherwise, the resulting weight matrices are used as initial conditions for experiments with frozen weights (*Δw*_*ij*_ = 0) for a duration of ${T}^{\exp }$ on which the analysis is performed.

Simulations: To complement the hardware emulations, an idealized version of the network is implemented in Brian 2^64^. Specifically, no parameter or temporal noise is considered, and weights are not discretized as it is the case for the neuromorphic chip. For simplicity, the degree of the input is implemented probabilistically by connecting each neuron-input pair with probability *K*_ext_/*N* and each pair of neurons with probability (*N* − *K*_ext_)/*N*.

### Evaluation

Binning: The following measures rely on an estimate of activity, therefore we apply temporal binning

10 $${\tilde{x}}_{i}(t)=\sum _{k}{\mathbb{1}}\left({x}_{i}^{k}\ge t\cdot \delta t,\ {x}_{i}^{k}\, < \, (t+1)\cdot \delta t\right)\ ,$$

where *δt* corresponds to the binwidth, and 1 is the indicator function. With this definition, we are able to define the binarized activity for a single process *i*

11 $${x}_{i}(t)=\min \left[1,{\tilde{x}}_{i}(t)\right]\ .$$

The variable *x*_*i*_(*t*) can represent either activity of a neuron in the network *a*_*i*_(*t*), or of a stimulus spike train *s*_*i*_(*t*), and correspondingly the spike times ${x}_{i}^{k}$ represent spikes of network neurons or stimulus (input) spike trains.

The population activity *a*(*t*) of the network is defined as

12 $$a(t)=\mathop{\sum }\limits_{i = 1}^{N}{\tilde{a}}_{i}(t)\ .$$

Neural avalanches: A neural avalanche is a cascade of spikes in neural networks. We extract avalanches from the population activity *a*(*t*), obtained by binning the spike data with *δt* corresponding to the mean inter-event interval, following established definitions. In detail, one avalanche is separated from the subsequent one by at least one empty time bin^43^. The size *s* of an avalanche is defined as the number of spikes in consecutive non-empty time bins. At criticality, the size distribution *P*(*s*) is expected to follow a power law.

To test for criticality, we compare whether a power law or an exponential distributions fits the acquired avalanche distribution *P*(*s*) better ^45^. For the fitting, first the best matching distribution is determined based on the fit-likelihood. The fit-range is fixed to *s* ∈ {4, 3 ⋅ *N*} as the system is of finite size. An estimation of the critical exponent *α*_*s*_ and an exponential cutoff *s*_cut_ is obtained by fitting a truncated power law

13 $${{\mathcal{P}}}_{{\rm{pl}}}(s)\propto {s}^{-{\alpha }_{s}}\exp \left(-\frac{s}{{s}_{{\rm{cut}}}}\right)\ ,$$

for *s* ≥ 1. Power law fits are performed with the Python package power law described in ref. ^65^.

Fano factor: The variability of the population activity is quantified by the Fano factor $F={\sigma }_{a}^{2}/{\mu }_{a}$, where ${\sigma }_{a}^{2}$ is the variance and μ_*a*_ is the mean of the population activity *a*(*t*), binned with *δt* = *τ*_ref_.

Trial-to-trial variability and susceptibility: The trial-to-trial distance *Δ*_VRD_ is obtained by stimulating the same network twice with the same Poisson spike trains, leading to two different trials *m* and *n* influenced by variations caused by the physical implementation. The resulting spike times in trial *m* emitted by neuron *i*, termed ${t}_{i,m}^{j}$, are convolved with a Gaussian

14 $${\tilde{t}}_{i,m}(t)=\sum _{j}{\int_{0}^{{T}^{\exp }}}\exp \left(-\frac{{(t-t^{\prime} )}^{2}}{2{\sigma }_{{\rm{VRD}}}^{2}}\right)\delta (t^{\prime} -{t}_{i,m}^{j}){\rm{dt}}^{\prime} \ ,$$

and likewise for trial *n*. The width is chosen to be *σ*_VRD_ = *τ*_ref_ and the temporal resolution for the integration is chosen to be 0.1 ms. From different trials *m* and *n* the distance is calculated

15 $${\Delta }_{{\rm{VRD}}}=\frac{1}{{\sigma }_{{\rm{VRD}}}}\sum_{{m,{\,} n}\atop{m {\,} \ne {\,} n}}\mathop{\sum }\limits_{i = 1}^{N}{\int_{-\infty }^{\infty }}\frac{{[{\tilde{t}}_{i,m}(t)-{\tilde{t}}_{i,n}(t)]}^{2}}{{\left[{\tilde{t}}_{i,m}(t)+{\tilde{t}}_{i,n}(t)\right]}^{2}}{\rm{dt}}\ .$$

To obtain an estimate of the networks sensitivity *χ* to external perturbations, a pulse of *N*_pert_ additional spikes is embedded in the stimulating Poisson spike trains at time *t*_pert_

16 $$\chi =\frac{a({t}_{{\rm{pert}}}+\delta t)-a({t}_{{\rm{pert}}})}{{K}_{{\rm{ext}}}^{2}}\ ,$$

normalized to the number of external connection ${K}_{{\rm{ext}}}^{2}$ to compensate for the decoupling from external input with decreasing *K*_ext_. The population activity is estimated with binsize *δt* = *d*_syn_. By evaluating *χ* immediately after the perturbation, only the effect of the perturbation is captured by minimizing the impact of trial-to-trial variations.

To calculate *χ* and *Δ*_VRD_, each weight matrix, obtained by the application of the plasticity rule, is used as initial condition for ten emulations with frozen weights and fixed seeds for the Poisson-distributed spike trains of duration *T*^pert^ and *T*^static^. In addition, a perturbation of size *N*_pert_ at *t*_pert_ = *T*^pert^/2 is embedded for the estimation of *χ*.

Autoregressive model: Mathematically, the evolution of spiking neural networks is often approximated by a first-order autoregressive representation. To assess the branching parameter *m* of the network in analogy to^42,43^, we make use of the following ansatz:

17 $$\langle a(t+1)| a(t)\rangle =m\cdot a(t)+h\ ,$$

where the population activity in the next time step, *a*(*t* + 1) is determined by internal propagation within the network (*m*), and by external input *h*. Here, 〈.∣.〉 denotes the conditional expectation and *m* corresponds to the branching ratio. For *m* = 1, the system is critical, for *m* > 1 the system is supercritical and activity grows exponentially on expectation (if not limited by finite-size effects), whereas for *m* < 1 the activity is stationary. The branching parameter *m* is linked to the autocorrelation time constant by ${\tau }_{{\rm{branch}}}=-\delta t/\mathrm{ln}\,(m)$. To obtain the activity *a*(*t*) the binwidth *δt* is set to the refractory time *τ*_ref_. Estimating *m* is straight forward here, as subsampling^66,67^ does not impact the estimate. Thus a classical estimator can be used, i.e., *m* is equal to the linear regression between *a*(*t*) and *a*(*t* + 1). For model validation purposes, the autocorrelation function *ρ*_*a*,*a*_ is calculated on the population activity *a*(*t*) binned with *δt* = *τ*_ref_

18 $${\rho }_{a,a}(t^{\prime} )=\frac{1}{{\sigma }_{a}^{2}}\mathop{\sum }\limits_{t = 1}^{{T}^{\exp }/\delta t-t^{\prime} }(a(t)-{\mu }_{a})(a(t+t^{\prime} )-{\mu }_{a})\ ,$$

where *σ*_*a*_ is the standard deviation, and μ_*a*_ the mean of the population activity. Subsequently, *ρ*_*a*,*a*_ is fitted by an exponential to yield the time constant *τ*_corr_.

Information theory: We use notation, concepts and definitions as outlined in the review^23^. In brief, the time series produced by two neurons represent two stationary random processes *X*_1_ and *X*_2_, composed of random variables *X*_1_(*t*) and *X*_2_(*t*), *t* = 1, . . . , *n*, with realizations *x*_1_(*t*) and *x*_2_(*t*). The corresponding embedding vectors are given in bold font, e.g., ${{\bf{X}}}_{1}^{l}(t)=\{{X}_{1}(t),{X}_{1}(t-1),...,{X}_{1}(t-l+1)\}$. The embedding vector ${{\bf{X}}}_{1}^{l}(t)$ is constructed such that it renders the variable *X*_1_(*t* + 1) conditionally independent of all random variables ${X}_{1}(t^{\prime} )$ with $t^{\prime} <t-l+1$, i.e., $p({X}_{1}(t+1)| {{\bf{X}}}_{1}^{l}(t),{X}_{1}(t^{\prime} ))=p({X}_{1}(t+1)| {{\bf{X}}}_{1}^{l}(t))$. Here, (⋅∣⋅) denotes the conditional.

The entropy (H) and mutual information (I) are calculated for the random variables *X*_1_ and *X*_2_, if not denoted otherwise. This is equivalent to using *l* = 1 above, e.g., H(*X*_1_) and I(*X*_1_ : *X*_2_) = H(*X*_1_) − H(*X*_1_∣*X*_2_). We abbreviate the past state of spike train 1 by ${{\bf{X}}}_{1}^{-}$: thus ${{\bf{X}}}_{1}^{l}(t-1)=\{{X}_{1}(t-1),X(t-2),...,{X}_{1}(t-l)\}$. The current value of the spike train is denoted by *X*_1_. With this notation the AIS of, e.g., *X*_1_ is given by

19 $${\rm{AIS}}({X}_{1})={\rm{I}}({X}_{1}:{{\bf{X}}}_{1}^{-})\ .$$

In the same way, we define the TE between source *X*_1_ and target *X*_2_

20 $${\rm{TE}}({X}_{1}\to {X}_{2})={\rm{I}}({X}_{2}:{{\bf{X}}}_{1}^{-}| {{\bf{X}}}_{2}^{-})\ .$$

The lagged mutual information for time lag *τ* is defined as ${{\rm{I}}}_{\tau }({X}_{1}:{X}_{2})={{\rm{I}}}_{\tau }\left({X}_{1}(t):{X}_{2}(t+\tau )\right)$. Integrating the lagged I defines the MC

21 $${\rm{MC}}({X}_{1}:{X}_{2})=\mathop{\sum }\limits_{\tau = 1}^{{N}_{\tau }}\delta t\left[{{\rm{I}}}_{\tau }({X}_{1}:{X}_{2})-{{\rm{I}}}_{{N}_{\tau }}({X}_{1}:{X}_{2})\right]\ ,$$

with a maximal delay *N*_*τ*_ = 100. The I of a sufficiently large *N*_*τ*_ is subtracted to account for potential estimation biases.

To access the information modification the novel concept of PID is applied^24,25,27^. Intuitively, information modification in a pairwise consideration should correspond to the information about the present state of a process only available when considering both, the own process past and the past of a source process. Therefore, the joint mutual information ${\rm{I}}({X}_{1}:{{\bf{X}}}_{1}^{-},{{\bf{X}}}_{2}^{-})$ is decomposed by PID into the unique, shared (redundant), and synergistic contributions to the future spiking of one neuron, *X*_1_, from its own past ${{\bf{X}}}_{1}^{-}$, and the past of a second neuron or an input stimulus ${{\bf{X}}}_{2}^{-}$: In more detail, we quantify

1. The unique information ${{\rm{I}}}_{{\rm{unq}}}({X}_{1}:{{\bf{X}}}_{1}^{-}\setminus {{\bf{X}}}_{2}^{-})$ that is contributed from the neurons own past.
2. The unique information ${{\rm{I}}}_{{\rm{unq}}}({X}_{1}:{{\bf{X}}}_{2}^{-}\setminus {{\bf{X}}}_{1}^{-})$ that is contributed from a different spike train (neuron or stimulus).
3. The shared information ${{\rm{I}}}_{{\rm{shd}}}({X}_{1}:{{\bf{X}}}_{2}^{-};{{\bf{X}}}_{1}^{-})$ that describes the redundant contribution.
4. The synergistic information ${{\rm{I}}}_{{\rm{syn}}}({X}_{1}:{{\bf{X}}}_{2}^{-};{{\bf{X}}}_{1}^{-})$, i.e., the information that can only be obtained when having knowledge about both past states.

I_syn_ is what we consider to be a suitable measure for information modification^27^.

The joint mutual information as defined here is the sum of the AIS and the TE

22 $${\rm{I}}({X}_{1}:{{\bf{X}}}_{1}^{-},{{\bf{X}}}_{2}^{-})={\rm{I}}({X}_{1}:{{\bf{X}}}_{1}^{-})+{\rm{I}}({X}_{1}:{{\bf{X}}}_{2}^{-}| {{\bf{X}}}_{1}^{-})\ .$$

We calculated H, AIS, I, and TE with the toolbox *JIDT*^68^, whereas the PID was estimated with the *BROJA-2PID* estimator^69^. The activity is obtained by binning the spike data with *δt* = *τ*_ref_ and setting *l* to 4 to incorporate sufficient history. I and TE as well as the PID were calculated pairwise between all possible combinations of processes. Results are typically normalized by H to compensate for potential changes in the firing rate for changing values of *K*_ext_ (Supplementary Fig. 1). For the pairwise measures, H of the target neuron is used for normalization.

Reservoir computing: The performance of the neural network as a reservoir^47,48^ is quantified using a variant of the *n*-bit parity task. The network weights are frozen (i.e., plasticity is disabled) to ensure that the network state is not changed by the input.

To solve the parity requires to classify from the network activity *a*_*j*_(*t*), whether the last *n* bits of input carried an odd or even number of spikes. The network is stimulated with a single Poisson-distributed spike train of frequency *ν* acting equally on all external synapses, i.e., the input spike times are ${s}_{i}^{k}={s}^{k}\ \forall \ i$. Spike times are binned according to Eq. (11) with binwidth *δt* to get a measure of the *n* past bits. The resulting stimulus activity *s*(*t*) is used to calculate the *n*-bit parity function according to

23 $${p}_{n}\left[s(t)\right]=s(t)\oplus s(t-1)\oplus ...\oplus s(t-n+1)\ ,$$

with ${p}_{n}\left[s(t)\right]\in \{0,1\}$ and the modulus 2 addition  ⊕ , i.e., whether an odd or even number of spikes occurred in the *n* past time steps of duration *δt*.

On the activity *a*_*j*_(*t*) of a randomly selected subset ${\mathcal{U}}$ of neurons with cardinality *N*_read_ a classifier is trained

24 $$v(t)=\Theta \left(\sum _{j\in {\mathcal{U}}}{w}_{j}{a}_{j}(t)-\frac{1}{2}\right)\ ,$$

where *Θ*(⋅) is the Heaviside function, and *v*(*t*) is the predicted label. The weight vector *w*_*j*_ of the classifier is determined using linear regression on a set of training data *s*_train_ of duration *T*^train^

25 $${w}_{j}=\arg \mathop{\min }\limits_{{w}_{j}}\left(\mathop{\sum }\limits_{t = 0}^{{T}^{{\rm{train}}}/\delta t-1}{\left|{p}_{n}\left[{s}_{{\rm{train}}}(t)\right]-{w}_{j}{a}_{j}(t)\right|}^{2}\right)\ .$$

The network’s performance on the parity task is quantified by ${\rm{I}}\left({p}_{n}\left[{s}_{{\rm{test}}}(t)\right],v(t)\right)$ on a test data set *s*_test_ of duration *T*^test^. The performance I is offset corrected by training the very same classifier on a shuffled version of *p*_*n*_[*s*(*t*)]. Moreover, we weighted each sample in the regression in Eq. (25) with the relative occurrence of their respective class to compensate for imbalance. Temporal binning with *δt* = 1 ms is applied to *s*_train_, *s*_test_, as well as *a*_*j*_(*t*).

In a second task, the stimulus activity *s*(*t*) is used to calculate the *n*-bit sum according to

26 $${z}_{n}\left[s(t)\right]=s(t)+s(t-1)+...+s(t-n+1)\ ,$$

i.e., how many spikes occurred in the *n* past time steps of duration *δt*. Here, the classifier described above is extended to multiple classes by adding readout units. The decision of the classifier is implemented by a winner-take-all mechanism across units.

In a third task, the readout is trained to calculate the NARMA system *x*_*n*_(*t*)

27 $$\begin{array}{l}{x}_{n}(t)=\alpha \cdot {x}_{n}(t-1)\\ \qquad\qquad\qquad\qquad\qquad\quad {\!\!\!\!\!\!}{\!\!\!}+\beta \cdot {x}_{n}(t-1)\cdot \frac{1}{n}\mathop{\sum }\limits_{i = 1}^{n}{x}_{n}(t-i)\\ \qquad\qquad\qquad\qquad\qquad {\!\!\!\!\!\!}+\gamma \cdot \tilde{s}(t-n)\cdot \tilde{s}(t-1)+\delta \ ,\end{array}$$

with *α* = 0.3, *β* = 0.05, *γ* = 1.5, *δ* = 0.1^70^, and the normalized stimulus activity

28 $$\tilde{s}(t)=\frac{s(t)-\min \left(s(t)\right)}{2\cdot \max \left(s(t)\right)}\ .$$

Again, a linear classifier is trained on the network activity

29 $$y(t)=\mathop{\sum }\limits_{j = 1}^{N}{w}_{j}{a}_{j}(t)\ .$$

Here, the performance is quantified by the normalized root-mean-square error (NRMSE)

30 $${\rm{NRMSE}}=\sqrt{\frac{{\left\langle {x}_{n}(t)-y(t)\right\rangle }_{t}}{{\sigma }_{y}}}\ ,$$

with the standard deviation of the vote of the linear classifier *σ*_*y*_.

## Supplementary information

Supplementary Information

Peer Review File

## Data Availability

Data available on request from the authors.

## Code availability

Code available on request from the authors.
