# Supplementary material for: Control of criticality and computation in spiking neuromorphic networks with plasticity
Source: Nat Commun. 2020 Jun 5;11:2853. doi: 10.1038/s41467-020-16548-3 (PMC7275091; doi:10.1038/s41467-020-16548-3)
Supplement: Supplementary file 1 — Supplementary Information [file 41467_2020_16548_MOESM1_ESM.pdf]

Supplementary Information for Control of criticality and computation in spiking neuromorphic networks with plasticity

Benjamin Cramer[1], David Stöckel[1], Markus Kreft[1], Michael Wibral[2], Johannes Schemmel[1], Karlheinz Meier[1], and Viola Priesemann[3,4,5]

[1] *Kirchhoff-Institute for Physics, Heidelberg University, Im Neuenheimer Feld 227, 69120 Heidelberg, Germany*
[2] *Campus Institute for Dynamics of Biological Networks, Georg-August University, Hermann-Rein-Straße 3, 37075 Göttingen*
[3] *Max-Planck-Institute for Dynamics and Self-Organization, Am Faßberg 17, 37077 Göttingen, Germany*
[4] *Bernstein Center for Computational Neuroscience, Georg-August University, Am Faßberg 17, 37077 Göttingen, Germany*
[5] *Department of Physics, Georg-August University, Friedrich-Hund-Platz 1, 37077 Göttingen, Germany*

April 15, 2020

Supplementary Figures

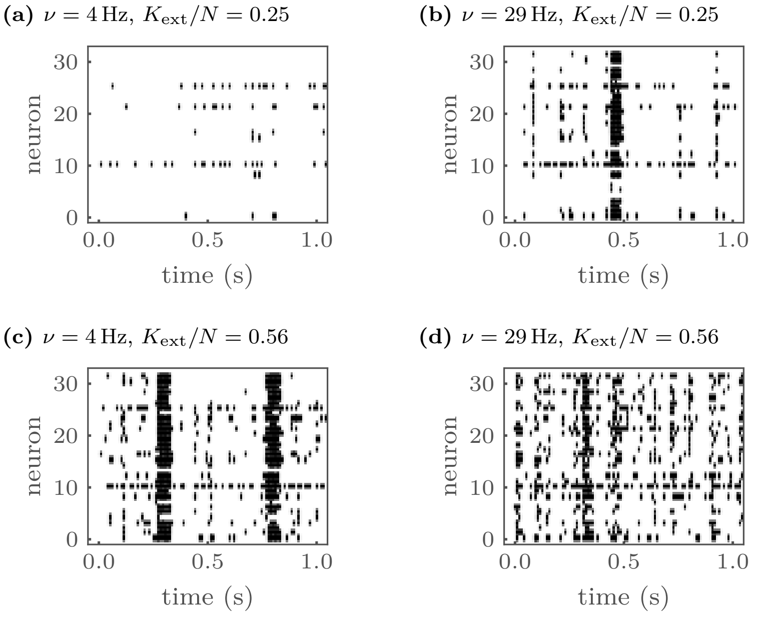

Supplementary Figure 1: The input strength shapes the collective dynamics of the network. This figure corresponds to Fig. 1 in the main manuscript. For every degree of the input K_{ext}, there is an input rate ν for which the activity shows irregular bursts, resembling a critical state. In the sub-critical case, the firing becomes more irregular and asynchronous. The input rate ν increases from left to right with $\nu = 4\,\mathrm{Hz}$ for (a) and (c) and $\nu = 29\,\mathrm{Hz}$ for (b) and (d). The degree of the input K_{ext} increases from top to bottom with $K_{\mathrm{ext}}/N = 0.25$ for (a) and (b) and $K_{\mathrm{ext}} = 0.56$ for (c) and (d).

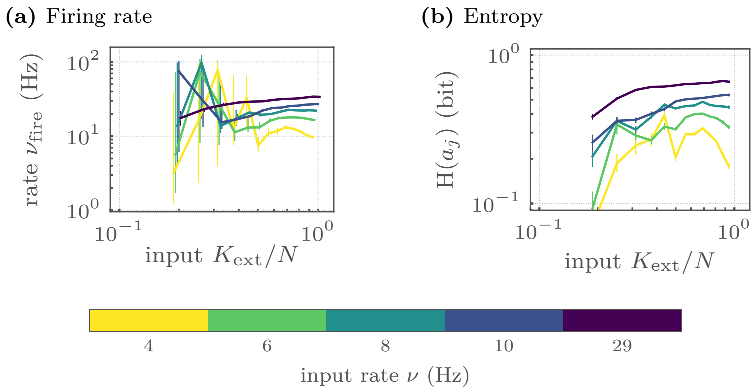

Supplementary Figure 2: Firing rates ν_{fire} and entropy (H) differ with input. (a) The degree of the input K_{ext} as well as the input rate ν affect the mean population firing rate ν_{fire}. (b) The entropy (H) of the spiking activity of a single neuron, a_j differ for various input strengths as a consequence of changing firing rates in (a), suggesting the need for normalization of information theoretic measures. In this and all following figures, the median over runs and (if acquired) trials is shown, and the errorbars show the 5%-95% confidence intervals.

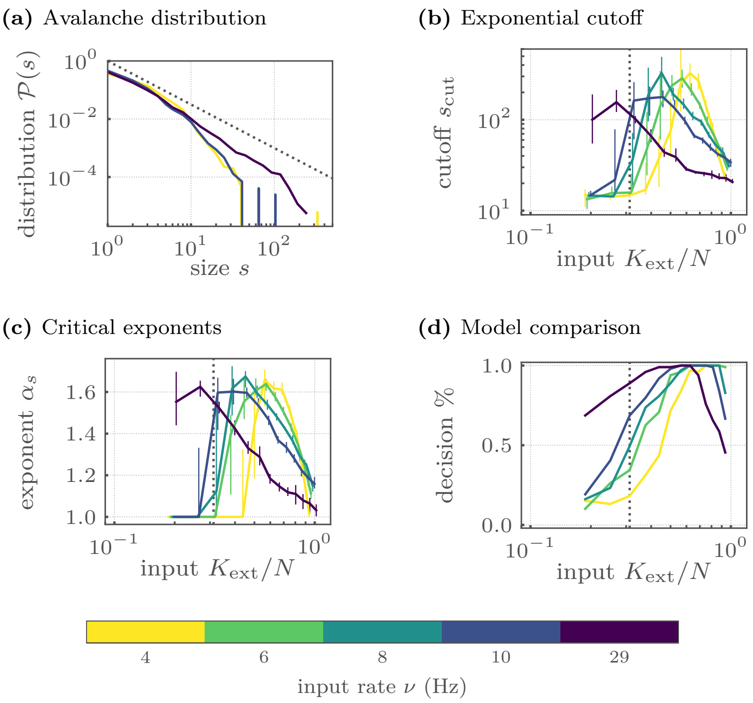

Supplementary Figure 3: Under specific input strengths, the network self-organizes towards a critical state, and shows long-tailed avalanche distributions. This figure corresponds to Fig. 2 in the main manuscript. The input strength is determined by both, the degree of external input K_{ext} and the input rate ν (colors). Only for specific combinations of these parameters, (a) power-law distributed avalanche sizes s over two orders of magnitude are observed (shown for $K_{\mathrm{ext}}/N = 0.31$). Fitting a truncated power law, (b) the exponential cutoff s_{cut} peaks, and (c) critical exponents α_s approximate 1.5 for the critical input strengths, as expected for critical branching processes. (d) A maximum-likelihood comparison decides for a power-law compared to an exponential fit in the majority of cases for the aforementioned critical input strengths. The dashed vertical line in (b) to (d) highlights the K_{ext}/N that has been selected in (a).

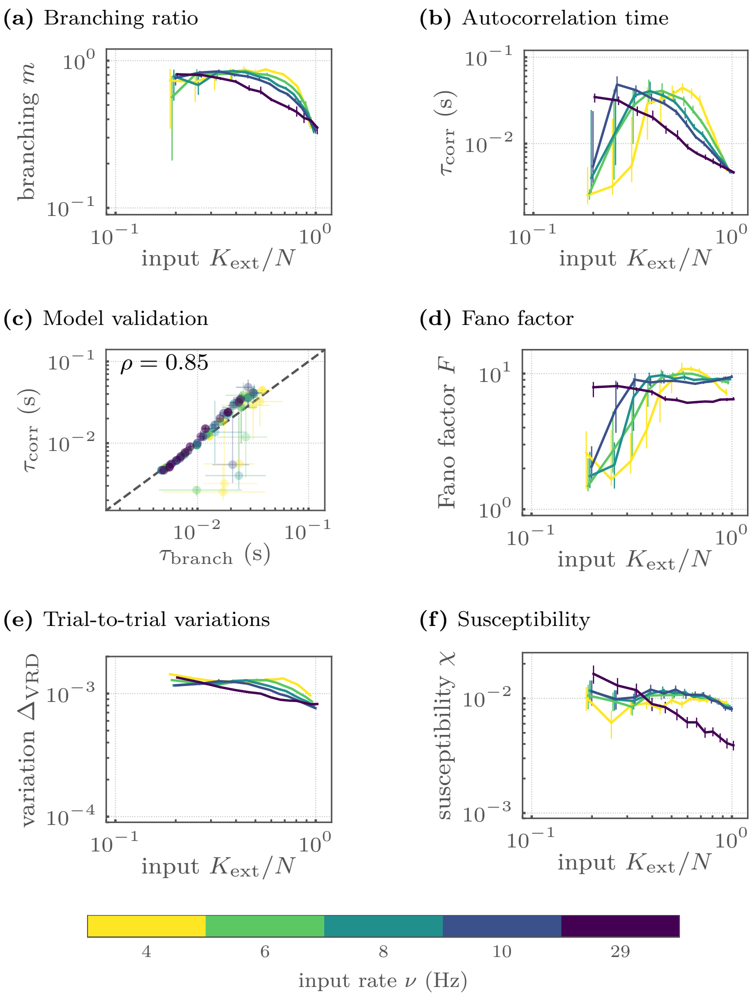

Supplementary Figure 4: Depending on the input strength, systems show clear signatures of criticality beyond power-laws. This figure corresponds to Fig. 4 in the main manuscript. The input strength is determined by both, the degree of external input K_{ext} and the input rate ν (colors). Only for specific combinations of these parameters, (a) the estimated branching ratio m tends towards unity, and (b) the estimated autocorrelation time τ_{corr} peaks. (c) The clear match of the τ_{corr}, and the $\tau_{\text{branch}} \sim -1/\log(m)$ as inferred from m supports the criticality hypothesis (correlation coefficient of $\rho = 0.850$, $p < 10^{-10}$). Further, intrinsic variations as measured by (d) the Fano factor F, and (e) the trial-to-trial variation Δ_{VRD}, as well as (f) to external perturbations as measured by the susceptibility χ peak approximately for the critical input strengths.

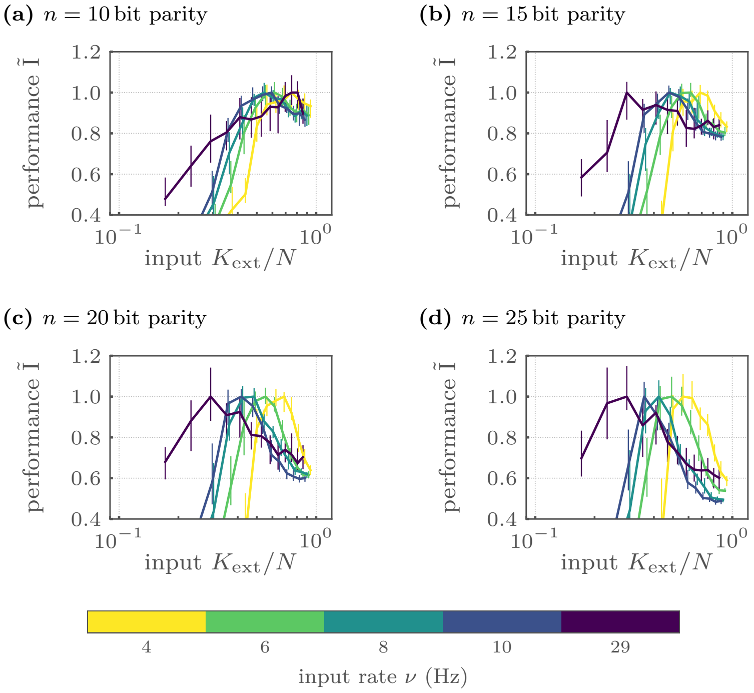

Supplementary Figure 5: Computational challenging task profit from critical network dynamics – simple tasks do not. This figure corresponds to Fig. 5a in the main manuscript. The network is used to solve a n-bit parity task by training a linear classifier on the activity of $N_{\mathrm{read}} = 32$ neurons. Here, task complexity increases with n, the number of past inputs that need to be memorized. Task-complexity n increases from (a) to (d) with $n \in \{10, 15, 20, 25\}$. For high n, task performance profits from criticality, whereas simple task suffer from criticality. The performance is quantified by the normalised mutual information $\tilde{I}$ between the parity of the input and the vote of a linear classifier. The performance $\tilde{\mathrm{I}}$ for high n-bit parity tasks is higher for the critical pairs of the external input K_{ext} and the input rate ν.

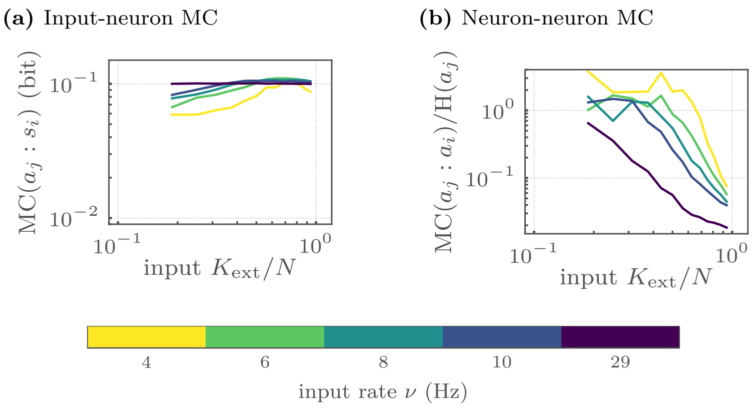

Supplementary Figure 6: Long lasting memory accompanies critical network dynamics. This figure corresponds to Fig. 6a and 6b in the main manuscript. (a) The memory capacity (MC) about the input s_i as read out by a neuron a_j stays almost constant. (b) In contrast, the memory capacity (MC) between pairs of neurons clearly peaks for the critical input strengths and saturates in the super-critical regime.

Supplementary Tables

**Parameter**	**Symbol**
Membrane potential of neuron j	u_j
Membrane capacitance	C_m
Membrane time constant	τ_{mem}
Leak conductance	g_{leak}
Threshold potential	u_{thresh}
Leak potential	u_{leak}
Reset potential	u_{reset}
Refractory period	τ_{ref}
Excitatory synaptic time constant	$\tau_{\mathrm{syn}}^{\mathrm{exc}}$
Inhibitory synaptic time constant	$\tau_{\mathrm{syn}}^{\mathrm{inh}}$
Synaptic delay	d_{syn}
Synaptic weight from neuron i to j	w_{ij}^{rec}
Synaptic weight from input i to neuron j	w_{ij}^{ext}
Synaptic current of neuron j	I_j
Weight conversion factor	γ
k-th spike time of input j	s_j^k
k-th spike time of neuron j	t_i^k
Number of neurons	N
Number of inhibitory neurons	N_{inh}
Number neurons available to classifier	N_{units}
Degree of the input	K_{ext}
Input rate	ν
Anticausal STDP time constant	τ_{stdp}
Anticausal STDP amplitude	η_{stdp}
Accumulation traces	f
Correlation scaling	λ_{stdp}
Drift parameter	λ_{drif}
Range of random variable	n_{amp}
Bias of random variable	$\langle n \rangle$
Noise at synapse from i to j	n_{ij}
Plasticity update period	T
Burning experiment duration	T^{burnin}
Static experiment duration	T^{exp}
Static trial experiment duration	T^{static}
Training experiment duration	T^{train}
Testing experiment duration	T^{test}

Supplementary Table 1: Overview of the model variables and parameters used in this manuscript.

**Parameter**	**Symbol**
Binwidth	δt
Neuron-wise activity	$a_i(t)$
Process-wise input activity	$s_i(t)$
Neuron population activity	$a(t)$
Input population activity	$s(t)$
Avalanche sizes	s
Avalanche size distribution	$\mathcal{P}(s)$
Critical exponent	α_s
Exponential cutoff	s_{cut}
Fano factor	F
Mean of population activity	μ_a
Variance of population activity	σ_a
Trial-to-trial variability	Δ_{VRD}
Width of convolution kernel	σ_{VRD}
j-th spike by neuron i in trial m	$t^j_{i,m}$
Convolved spike train of neuron i in trial m	$\tilde{t}_{i,m}$
Susceptibility	χ
Perturbation size	N_{pert}
Perturbation time	t_{pert}
Branching ratio	m
External input	h
Autocorrelation function	$\rho_{a,a}$
Autocorrelation time constants	τ_{branch}, τ_{corr}
Stationary random process j	X_j
Random variable	$X_j(i)$
Realisation of random variable j	$x_j(i)$
Embedding vector of dimension l	$\mathbf{x}^l_j(i)$
Current state of process j	$\mathbf{x}_j$
Past state of process j	$\mathbf{x}^-_j$
Information Entropy	$\mathrm{H}(x_j)$
Mutual information	$\mathrm{I}(x_j : x_i)$
Conditional mutual information	$\mathrm{I}(x_j : x_i \vert x_k)$
Joint mutual information	$\mathrm{I}(x_j : x_i, x_k)$
Active information storage	$\mathrm{AIS}(x_j)$
Transfer entropy	$\mathrm{TE}(x_i \rightarrow x_j)$
Lagged mutual information	$\mathrm{I}_\tau(x_j : x_i)$
Memory capacity	$\mathrm{MC}(x_j : x_i)$
Number of delay steps	N_τ
Unique information	$\mathrm{I}_{\mathrm{unq}}(x_j : x_i \setminus x_k)$
Shared information	$\mathrm{I}_{\mathrm{shd}}(x_j : x_i; x_k)$
Synergistic information	$\mathrm{I}_{\mathrm{syn}}(x_j : x_i; x_k)$
n-bit sum function	z_n
n-bit parity function	p_n
n-bit NARMA function	x_n
Weight form neuron j to readout	w_j
Vote of classifier	$v(t)$
Training data	s_{train}
Testing data	s_{test}
Normalized stimulus activity	$\tilde{s}(t)$
Normalized mutual information	$\tilde{I}$
Normalized root-mean-square error	NRMSE

Supplementary Table 2: Overview of the evaluation variables and parameters used in this manuscript.

Supplementary Notes

In the main part of this manuscript, the distance to criticality has been controlled by the degree of the input K_{ext}. K_{ext} is only one possibility to control the input strength of a neural network. Indeed the input strength could also be adjusted by the input rate ν. In conjunction, K_{ext} and ν shape the network response where only certain parameter combinations allow for the observation of signatures of criticality. In this supplementary information, we show that tuning ν has qualitatively the same impact as tuning K_{ext}. The Supplementary Figures 1, 3, 4, 5 and 6 show the same results as in the main text, but for varying K_{ext} and ν.

In addition, an overview of all parameters and variables is given in the Supplementary Tables 1 and 2.
