# Supplementary material for: Control of criticality and computation in spiking neuromorphic networks with plasticity
Source: Nat Commun. 2020 Jun 5;11:2853. doi: 10.1038/s41467-020-16548-3 (PMC7275091; doi:10.1038/s41467-020-16548-3)
Supplement: Supplementary file 2 — Peer Review File [file 41467_2020_16548_MOESM2_ESM.pdf]

Reviewers' comments:

Reviewer #1 (Remarks to the Author):

The manuscript “Role of ferroelectric polarization during growth of highly strained ferroelectrics revealed by in-situ x-ray diffraction” by Rui Liu et al. presents a significant contribution to our fundamental understanding of the role of electric polarization in the growth process of epitaxial thin films. This work helps to fill a gap in studies of complex oxide multilayer growth and properties as the contribution of polarization coupling effects is often overlooked.

The central claim of the manuscript is that the electric polarization state of an oxide layer (lead titanate, PTO) affects the growth rate, relaxation, and polarization domain structure of the layer (barium titanate, BTO) that is grown on top of PTO. The ferroelectric polarization state of PTO layers is controlled by PTO film thickness and temperature before BTO layer deposition. As coincidence does not imply causation, it has been important for the authors to present strong arguments that the observed changes in structure and properties of BTO films are caused by PTO polarization and not by temperature or strain coupling. I think that experimental data presented along with thermodynamic theory modeling are sufficient to support the main claim of the article. The weaknesses of the manuscript in my opinion are that (1) the work is narrowly focused on one system only with little or no discussion of the applicability of findings for other systems and materials and (2) modeling does not include electrostatic interactions between materials explicitly.

I believe this work can be important and of great interest to a broad range of fundamental science and materials engineering researchers. I can recommend this manuscript for publication in Nature Communications provided the authors consider the following questions and comments:

1) The manuscript in its present form is very narrowly focused on one system, BTO/PTO, and broader impacts of this research are briefly mentioned only at the very end of the article. I think it is important to consider other ferroelectric/ferroelectric systems in which the authors expect to find similar polarization-controlled growth effects. Also, I suggest the significance of this research to broader audience in the field of thin-film research is emphasized more at the beginning of the paper (and/or in the abstract).

2) What is the origin of polarization-controlled growth? Generally, the film growth depends on the surface and interface energies. It could be useful to estimate the changes of the surface energies with polarization (and temperature) instead of using the Gibbs free energy.

3) Can we expect to find similar polarization-controlled growth effects in multilayers including non-ferroelectric layers? STO on PTO? ZnO on PTO?

4) The authors assume for thermodynamic theory calculations that the spontaneous polarization of the PTO layer can be imposed on BTO and used to analyze the stability of the BTO layer that grows on PTO. In fact, it is more likely that polarizations in both BTO and PTO layers will be different from their thermodynamic equilibrium values of pure substances due to electrostatic polarization coupling. The total free energy of the system will be the sum of G(BTO), G(PTO), and an extra energy term due to electrostatic polarization coupling (see, for example, Okatan et al. PRB 79, 174113 (2009)). Ideally, and this is especially important when the film thickness increases, the authors should consider adding coupling to their calculations and present the total free energy of the multilayer system instead of the energies of individual non-interacting layers.

5) It has been predicted that the electrostatic coupling is weaker in thicker ferroelectric layers. For instance, a transition from strong to weak coupling in BTO/PZT (Salev et al. PRB 93, 041423R (2016)) in the range of film thicknesses between 10 nm and 30 nm leads to different polarizations (close to single-layer polarization values) in different layers. Could it be that the BTO layer relaxation transition in films thicker than 25 u.c. (Fig. 6 in the manuscript) is related to the

coupling transition that can only occur when the substrate (PTO) is ferroelectric?

Reviewer #2 (Remarks to the Author):

This is a very interesting paper that describes experiments demonstrating that the ferroelectric polarization of thin PbTIO3 layers can have a significant effect on the growth behavior and subsequent room T properties of BaTiO3. The authors demonstrate convincingly that the polarization magnitude of the PbTiO3 layer is responsible for this interesting behavior. Although I see one problem with the paper (described in the following), overall I feel that the study presents interesting and potentially important information of broad general interest. Thus, I strongly support publication of the paper in Nature Communications after editing to address the following issue.

The one concern I have is that the model described starting in the bottom paragraph of page 9 indicates that stripe domains in the PbTiO3 layer play a significant role in controlling the domain structure of the BaTiO3. They cite a paper by Fong et al. (ref 9) as evidence that the thin PTO layers form 180° stripe domains. However, it should be noted that the results described in ref. 9 were for films grown directly on SrTiO3 substrates. Subsequent studies by that same group found that films grown on conducting SrRuO3 layers on STO are monodomain, since the SRO layer provides electrical compensation at the lower PTO interface (see Fong et al. PRL 96, 127601 (2006)). Since the PTO layers in the current study were grown on conducting SRO layers, they are very unlikely to have 180° stripe domains. The authors need to modify the manuscript to take this into account.

Reviewer #3 (Remarks to the Author):

Review of Liu et al,
"Role of ferroelectric polarization during growth of highly strained ferroelectrics revealed by in-situ x-ray diffraction"

The primary result of this paper is the intriguing observation that if BaTiO3 (BTO) is sputter-deposited on a polarized interface, the growth rate unexepectedly is lowered in a systematic way based on the polarization strength at the surface. If the surface is tuned to be non-polar, then the growth rate is constant as expected. As noted by the authors, this points towards exciting additional ways to engineer ferroelectric thin films materials properties during growth.

I agree that this is an interesting and novel observation and would be of interest to others in the community, although I apologize in advance to the authors that I do not recommend publication of the paper in its current form.

The authors performed a set of experiments that tuned the polarization strength at the surface by using ferroelectric properties of specially prepared heterostructure substrates created with a ultrathin layers of coherently strained PbTiO3 (PTO) on top of 20nm SrTiO3 epitaxial on top of SrTiO3 (100) crystal substrate. At a range of temperature around the typical growth temperatures for the BTO, the PTO undergoes a phase transition from paraelectric or ferroelectric, and the magnitude of the polarization in the ferroelectric phase depends on temperature and thickness of the underlying PTO film.

The authors show a plausible and reasoned argument that suggests this unanticipated effect is not due to details of sputter deposition process (such as the changing proportion of neutral/ionized BTO subspecies with sputtering conditions) but might be explained using a thermodynamic argument based attachment of BTO unit cell (uc) if it is required to be in a polarized versus un-

polarized state.

In addition, there are several secondary complications which they also documented during their experiments. These are all related to the significant strain relaxation unavoidably occurring during their depositions as the BTO film becomes thicker than about 10-15 nm. I note that strain relaxation could be expected to have an effect on growth rate, although one might assume that deposition is 'easier' when the film is relaxed closer to the bulk lattice parameters than when species are trying to maintain a coherently strained lattice. If this had an effect on growth rate, it would be opposite to what is observed in this paper.

They observe that the strain relaxation in the BTO film growth is suppressed somewhat for a BTO film grown on a polarized surface, coincident with the growth mode of BTO presenting a more smooth or layer-by-layer aspect on the polarized surface, but the growth mode is rather rough on the un-polarized surface. (See Supplemental Figure 6 and 7) The authors note that these two aspects are correlated and also suggest that the PTO polarization (which is expected to enhance tetragonality in the BTO) also helps the BTO maintain coherency for slightly thicker films.

I am very interested in the results and their interpretations.

Unfortunately, while I believe the authors have a careful and systematic set of experiments and have thoughtfully combined many techniques and supporting characterizations that may provide a complete picture to themselves, I also had a overly hard time parsing through this paper due to its limitations.

The presentation of the work is disorganized and in places, sloppy. I do not recommend the publication of this work in its current form. I suggest that the authors take the time to tighten up the organization, check their statements for precision and clarity, check their figures and descriptions of their methods for accuracy.

While the following is not exhaustive, I have outlined some of the issues that I puzzled over. If I have misinterpreted some of the experiments, that is consistent with the issues I had in working with this paper in its current form.

A) What are the implications or issues of measuring a growth rate that may be varying over time?:

Fig 1 shows the primary proof of the assertion of the BTO growth rate decreases with increasing surface polarization. As far as I can tell from the paper, I assume that the growth rates must have been (?) determined by sputter depositing an approximately 20nm BTO film, then knowing the time and measuring the thickness via XRD (Supplemental Fig 1) gives the growth rate.

This protocol would give the *average growth rate* over 50 unit cells of the 20 nm BTO film (from coherency to partically or fully relaxed). Note that different systems shown spend different amounts of time in various stages of relaxation that depends on the thickness of PTO underlayer and the growth temperaure.

Consider the comparison growth 'rate' for the 50nm BTO/PTO superlattice on SRO(?)/STO. I would assume that the 1st 3 unit cells of BTO grown on the 1st 3unit cells of PTO on the 'virgin' SRO/STO substrate), should act *identically* (SAME GROWTH RATE) to the 1st 3 unit cells of the 20nm BTO film grown on a 3uc PTO/SRO/STO substrate. The average growth rate, however, as documented in the figure, ends up completely different.

In the subsequent synchrotron experiments, the authors note that they measured the growth rate from the CTR oscillations at the BTO (0 0 1/2) position (Supplemental Fig 6). This could distinguish the rate for the 1st few unit cells (before significant relaxation) and isolate it from an average.

Were the in-situ synchrotron xray measurements consistent with the growth rates and trends shown in Figure 1? In particular, what are the rates for the first few BTO unit cells before relaxation complicates things?

It is possible that these calibration growths were only done for a few samples (in order to set the sputtering conditions to get roughly the same growth rate in the new chamber as the in-lab experiments?). Perhaps the desired data doesn't exist, but that wasn't clear from the descriptions of methods.

B) Supplemental Figure 1 - the x-axis is not labeled (sloppy):
It is noted that the plot is from theta/2theta XRD scan, but is the x-axis listing the theta or twotheta values? Also why not label some of the features - I assume the peak is a resolution broadened STO(?) 001. I calculated fringe oscillations on this curve that the SRO and the BTO films are each closer to 10nm rather than the 20nm stated, but I could be mistaken.

C) Inconsistency in domain size in AFM images and the x-ray results (micron compared to 30nm domains):
Figure 3 shows piezo-AFM, with ferroelectric domain sizes being micron or sub micron. Diffuse scattering in Figure 4 shows domain sizes in these films should be approximately 20-60nm, or basically about 30nm at end of growth.
1) Why are there only sub-micron/micron size domains in the p-AFM and no sign of the finer domain structure?
2) If the domains coarsen that significantly when the samples are cooled, is this consistent with the x-ray results? (as the samples are cooled further, if the domains coarsen, the diffuse scattering would crawl back under the CTR. Was this observed?

D) Inconsistency in the calculation of the domain size in Figure 4:
Although the images are highly saturated in order to help show the diffuse scattering, in Figure 4a) the 3uc 300C image shows clear maxima in the diffuse scattering at about 0.01 inverse angstroms from the CTR. This is a periodicity of about 60 nm (600 angstroms = 2pi/.01). Assuming diffuse peak is from correlations from ~1up/~1down domain periodicity, then each up or down domain itself about 30nm. These images are on 10nm thick films, or about 25 unit cells. However, In Figure 4b, it suggests the automatic fitting procedure of the domains gave a domain size of 25nm which seems low compared to the image even despite the graphical estimations I did on the figures.

To help with showing more features in the colormap, plot log(I) instead of Intensity. If intensity colormap is log(I), then that should be noted explicitly, right?

E) Inconsistency in Supplemental Figure 7 (sloppy?):
In supplemental figure 7a and b, the curves that are shown by the red dots in a and b should be identical since they are the identical sample [4uc PTO at 540C]. They are not identical. Is this in error? If these are nominally identically prepared samples but two different experimental runs, then it also gives some indication of reproducibility and should be taken into account in other aspects of the work.

F) (unclear or rationale unclear):
In the preparation of the underlying heterostructures (n uc PTO/20nm SRO/STO(100) it was implied that each ultrathin layer of PTO was grown at different temperatures (based on its calculated Tc?) ("ultrathin PTO films were grown at temperatures in the vicinity of the ferroelecric phase transition temperature") , which would be a very odd temperature to grow the PTO underlayer rather than creating identically grown heterostructures and only varying the subsequent temperatures in the BTO growths, however, perhaps I didn't understand the rationale or misinterpreted the statement.
Wouldn't using similar temperature and conditions be rather critical in creating as similar a surface

morphology of the ultrathin PTO?

G) Supplemental Figure 1 as shown adds to reader confusions rather than illuminates:
This figure showing the CTR's for 3 samples grown to approximately 20nm. There is no significant difference in total final thickness and thus growth rate is roughly constant, despite the fact that they cover the three temperatures below/at/above the calculated TC of the 3uc PTO underlayer substrate. Initially this figure confused me as being inconsistent with the authors claims that the phase transition would affect the rate. Then I noticed that Figure 1 in the paper similarly shows NO significant change in growth rate for this particular sample at those particular temperatures).

Wouldn't it be useful to add one further temperature for comparison or show different sample that actually has a growth rate difference to help reader visualize the experiments better.

Or explain to reader more clearly what they are supposed to be learning or visualizing from this figure.

Reviwer #2

The major claim of this manuscript is that computational performance of recurrent neural networks is not necessarily maximized at their critical state, as opposed to the often reported cases. Through the investigation of the temporal parity check problem where the task complexity can be changed, the authors show that the criticality is beneficial for complex computational tasks but not for simple ones. After demonstrating that the distance to the critical point can be controlled by the single parameter (meaning the level of external inputs K_{ext}) in the small recurrent neural networks emulated with the neuromorphic device (BrainScaleS 2), the relationship between the dynamical behavior of the network (i.e. how close to the critical state) and the task performance is examined. Further the task-independent information-theoretic measures are used to characterize the computational properties of the recurrent neural networks. This manuscript deals with the challenging topic (i.e. the relationship between dynamics and computational ability in neural networks), which may attract much interests particularly from the communities of nonlinear physics, neural networks, and neuromorphic hardware. The methods are technically sound and the texts are well written.

We would really like to thank the reviewer for the interesting and valuable comments which significantly improved our manuscript.

Major comments:

1. *The claim of this manuscript is based on the results for the specific task (the parity check problem). The complexity of this task is mainly related to the length of the memory in the computational model, but other factors such as nonlinearity are also involved in task complexity in general. To deduce the general statement as in this manuscript, other benchmark tasks for evaluating the performance of reservoir computing models should be tested and consistent results should be shown. In other words, I feel that the claim is too generalized for the given computational results.*

   We thank the reviewer for this valuable suggestion. We added two types of tasks: One which is more simple than the non-linear parity task, namely a sum task (Fig. 5a). The other one is more challenging because the goal is to read out the parity/sum from a very small set of neurons (Figs. 5c and 5d). These two tasks are the most reasonable extensions we could come up with. Keeping in mind that both, the input as well as the activity in the network are binary, classical non-linear tasks like NARMA are not meaningful. Therefore, the parity task seems to be the most general non-linear task. We could only make it more difficult by restricting the input to the readout neurons. We could not imagine a task with precise and continuous control of non-linearity operating on binary inputs, but would welcome any ideas by the reviewer.

   To detail the two new tasks, it turns out that especially non-linear tasks profit from critical dynamics. Further, the non-linear information about the stimulus is only globally available in the network response if dynamics are critical. This nicely demonstrates how spatio-temporal correlations introduced by critical-like dynamics can be exploited for actual computation (lines 252–304).

2. *The significance of the use of the emulator with the existing neuromorphic circuits is not clear. The same experiments can be done using software simulations. In fact, some results (Fig.3) are based on software simulations. In addition, the readout from the emulator outputs (linear regression in the parity check task) is done using software computation. What is the essential merit of the use of the emulator only in a part of the whole computational system? If thermal noise is essential, how does it affect the results?*

The presented experiments can in principle be performed using software simulations. In the control software simulations, we checked the validity of our hardware implementation and the ability to scale to larger system sizes. However, the neuromorphic chip is about a factor 100 faster when using only $N = 32$ neurons. To give numbers, a single plasticity experiment with a duration of 600 s biological time is simulated in 570 s in Brian 2, but emulated in only 6 s on the neuromorphic chip (including IO and network initialisation). We added these number to our manuscript (line 193–207) to underpin the benefit in terms of emulation speed. Apart from this speedup, the implementation on the neuromorphic chip is a major breakthrough itself. This breakthrough is important scientifically, because when running such detailed networks as classical simulations, the computational overhead scales with $O(N^2)$ due to the all-to-all connectivity and synaptic plasticity on conventional hardware. For the neuromorphic system, the execution time is independent of the system size N (thus constant), as long as the network fits on the chip. For details we referenced the work of Wunderlich et al. 2018 in the lines 132–135 in the main manuscript. In this work, the power consumption as well as the execution speed is quantified.

In general, the readout could also be implemented on the neuromorphic chip by making use of the plasticity processor. This would however involve major effort (e.g. fixed-point precision) which we would like to postpone to future projects.

Noise seems to be not that essential when comparing the results obtained with the neuromorphic chip and with software simulations for $N = 32$. On the one hand, thermal as well as parameter noise put stability at risk and have therefore to be compensated. On the other hand, both forms of noise might help to avoid pathological synchronization of neural firing. The investigation of the impact of noise especially on larger systems is an interesting research topic that we are currently following up.

3. *I have a concern about a lack of quantitative correspondence between the information-theoretic measures and the computational performance. The information-theoretic measures change approximately monotonically as in Fig.6, but the task performance does not as in Fig.5(a)-(b). It seems to be difficult to tune the input K_{ext}/N at the optimal value for maximizing the computational performance only from the analysis of the information-theoretic measure. For a given task with a given complexity, what is the strategy to set the optimal value of input K_{ext}/N?*

   There are two aspects for considering information theory. First, criticality has been claimed to maximize information capacity. Indeed, we find that our networks enfold their maximum capacity at the critical point (e.g. Fig. 8). Second, it seems to be natural to use information theory to translate task-dependent performance. Here, the lagged mutual information between neuronal activity and the stimulus is informative for the transition from task-independent to task dependent considerations. From Fig. 7, time-scales could be inferred: To solve the 5-bit parity task, the network needs to memorize 5 ms of the input where the highest K_{ext} shown in Fig. 7a causes highest information about the input. In contrast, the 25-bit parity task requires memory of about 25 ms. Here, the network with lowest K_{ext} in Fig. 7a leads to highest information. We rewrote the manuscript accordingly (lines 104–111 and 510–515).

Minor comments:

1. *Page 2, model overview. What is the reason for using the small-scale ($N = 32$) recurrent neural networks? Is there any physical constraint of the emulator or is it possible to perform the same emulation with a larger-scale system? It is better to mention this point.*

   In brief the system has only 32 LIF neurons. In detail, due to the analog implementation, the number of neurons is limited to the number of available neuron circuits. We tried to clarify this by rephrasing the first sentence in the model overview paragraph in the lines 114–117. Future version of the chip will feature 512 neurons.

2. *Page 3, left column. The text mentions figure 2a and then figures 4a, 4b, and 4d. It is a little bit confusing. The indication of figures should be in the order of appearance.*

   We agree with the reviewer that the figure numbering is confusing at this point. We accordingly referenced the figures 2 and 3 as a whole in the lines 150–155 as both of them cover the analysis of neural avalanches and the level of detail is not required.

3. *Page 4, left column. The reference [50] is not called liquid state machine. It is called echo state networks. This citation is misleading. It is also better to mention physical reservoir computing.*

   We are aware of the clash of nomenclatures. We had stuck to the nomenclature of Wolfgang Maass, but would also prefer more neutral terms. Therefore, we replaced *liquid state computing* with *reservoir computing*.

Overall, I feel that this work is worth publishing after revisions taking into account the above comments.

Reviewer #3

In this paper, the Authors investigate the relation between criticality and task-performance. To achieve some simulations, they implemented a SNN in hardware neuromorphic chip. They performed several simulations showing that simple tasks are suffering from criticality. Tuning the input force, the system can be closed to criticality or far depending the complexity of the studied tasks. This paper is well written and clear to read. However the main thesis of this paper is to explain that criticality is not good for simple tasks. This is quite interesting and then a tuning of the state of the network depending applications is a good improvement. However, in my opinion, I am not sure this conclusion is a big ground-break in this field.

It is a common belief that criticality is beneficial for information processing. We clearly show that this is not the case for simple tasks, although criticality maximizes abstract computational properties. As this belief is common in neuroscience and other fields, we think that our work is of interest for the broad readership of Nature Communications.

To strengthen our point further, we added additional tasks and also showed the dynamics of switching the working point of our networks as suggested by the reviewer.

I have several comments:

1. *As you use BrainScaleS 2 prototype which can perform high number of neurons, why are you using just 32 neurons? In other simulations, why are you using Brian 2? And not keeping on with BrainScaleS 2? You wrote: as the physical system at hand is of limited, what is the limitation? It could be better to use the same tool for all experiments and also to enlarge the size of the simulated network. In discussion part, you said that analog neuromorphic devices (like BrainScaleS) have some internal noise. In that case, the simulation of the 32 neurons in hardware and simulations should present some differences (even minor ones). Could you compare them? To summarize, I do not understand in this work the need of a hardware neuromorphic system as you work with few number of neurons and you don't need real-time or accelerated time.*

   In brief, getting the neuromorphic chip to run stably is a achievement in itself. Our results will be highly valuable for future chips.
   First, we would like to clarify the hardware constraints: Currently, high neuron counts are only available for the BrainScaleS 1 system which lacks a plasticity processor. The neuromorphic chip used in this work is a small prototype of the BrainScaleS 2 platform with only 32 LIF neuron circuits. A full size version with 512 LIF neurons will be available in the future. In order to clearly delimit from the large BrainScaleS 1 system, we added a photograph of the small prototype setup in Fig. 1a and a footnote on page ii containing information about the future system.

   Checking for finite-size scaling is a basic requirement in any criticality analysis. To perform finite-size scaling with more than $N = 32$ neurons, we had to utilize software simulations (Brian 2). Moreover, these simulations served as control experiments to validate our hardware implementation. We tried to clarify this by revising the lines 180–182 in the main manuscript.

   In our setting, the accelerated time has greatly helped us to collect large amounts of data timely. We needed large amounts of data for two reasons: First, information theory requires sufficient statistics. Second, for the long-lasting adaptation phase of critical systems real-time emulation would be

lengthy. To give numbers, a single plasticity experiment with a duration of 600 s biological time is emulated in only 6 s on the neuromorphic chip (including IO and network initialisation). Further, we used the aforementioned neuromorphic chip as a case study for possible usage scenarios of noise affected analog neuromorphic systems. We included a paragraph dealing with the execution time in the lines 193–207 of the main manuscript.

The results from the neuromorphic chip and the software simulations show only minor differences for $N = 32$; the estimated cutoff parameter and the critical exponents are comparable. Thermal, as well as fixed-pattern noise might be helpful to avoid pathological synchronization of neural firing. The investigation of the impact of noise especially on larger systems is an interesting research topic that we are currently following up.

2. *You said that with low degree of input, the network is closed to criticality. However on Figure 2 d) it seems that with very low input the % decision decreases. Could you explain it? Same questions for Figure 5 a) and b) with low input K_{ext}/N*

   We thank the reviewer for the valuable comment. For low values of the input K_{ext} the networks tend to get unstable most likely due to the small system size. Because of this, we were not able to get closer to the critical point. Alternatively, the distance to criticality could equally be controlled by scaling the input rate where we observe a similar behavior, but for different values of K_{ext} (cf. supplementary material, Figs. 3 and 4). Here, the characteristic decrease when crossing the critical point can also be observed. We added this information in the lines 174–177 in the manuscript.

3. *You are using LIF neurons, do you have similar results with more complex neurons? As Brainscale 2 can simulate multi-compartmental neurons, it could be nice to try if the neural dynamics change and if criticality are similar. Why did you choose this number of neurons?*

   Indeed, it would be interesting to look at more complex neuron models with additional mechanisms as adaptation or multi-compartment features and their effect on critical-like dynamics. However, the used prototype lacks the aforementioned features and implements LIF neurons. As Adex (adaptive exponential) neurons with multi-compartment option are only scheduled for the full-size BrainScaleS 2 chip, we had to stick to the LIF neuron model.

4. *About STDP influence in the discussion part, how could you improve this part as full STDP (depression and potentiation) is useful for a lot of applications?*

   Actually, our rule features both, depression by anticausal spike pairings as well as unspecific potentiation. However, the potentiation is implemented by a positive drift of synaptic weights and not by causal spike pairings. Standard STDP causes a bimodal weight distribution and thus instabilities. We think that our rule represents an elegant way to circumvent the aforementioned observation as we have depression and unspecific potentiation. However, it is not fully clear yet whether one of the STDP variants has advantages in terms of applications. We clarified that by adding underbraces to Eq. 7 in the methods, specifying each term.

5. *For neural avalanche, how do you choose the bin size (δt)? Reducing or increasing it have influence on the results?*

   The bin size is an important parameter for the evaluation of neural avalanches and should be mentioned in the result section. Specifically, we stick to the common choice, and use the mean inter-event interval. We added this detail directly to the result section in the lines 164–167. The concerns of the reviewer

regarding the binsize are absolutely right, the dependence of the distribution of neural avalanches on the binwidth is an intrinsic problem. Because of this, we had tested also different binsizes and found similar results. More importantly, we considered all the other measures which are more robust (Wilting & Priesemann 2018). We decided to still include the distribution of neuronal avalanches as standard method to quantify the distance to criticality.

6. *You analyze all data after STDP. What happens if simulations are done at beginning of STDP process, middle and end? Is there an influence on the criticality following the STDP rule and then on the performance?*

   The investigation of the temporal evolution is an interesting research direction. We decided to include the case study proposed by the reviewer in comment 7, where we switch the degree of the input and monitor dynamics. We would like to refrain from an analysis starting from an initial state where all synaptic weights are zero as the activity is rather epileptic before convergence of synaptic weights (mainly due to discrete synaptic weights and device mismatch). For these dynamics, many of the considered measures are ill-defined. The network activity is much more well behaved when switching the degree of the input.

7. *In my point of view, this paper can be improved adding one case study. For instance solving two different tasks (one simple, one complex) with the same SNN but with tuning parameters and input strength (to switch from criticality or not) during the simulation.*

   We included a case study where we switched K_{ext} for a single SNN and evaluated task performance after various update steps (Fig. 6). In more detail, we selected two K_{ext} leading to good performance for either the simple or complex parity task and switched between them. As implementation, we only thought about a hierarchy of networks, each of which tuned to a different K_{ext}, but really liked the idea by the reviewer and now discuss it as well (lines 305–331).

   Moreover, we added two further types of tasks. For details we refer to comment 1 of Reviewer #2.

REVIEWERS' COMMENTS:

Reviewer #2 (Remarks to the Author):

The authors almost appropriately revised the manuscript according to the comments from the reviewers. In particular, the additional results on the computational performance under temporally switched inputs are interesting.

The two newly added tasks are not so complex compared with other practical temporal tasks. Any non-binary information can be in principle coded by binary information at a certain precision, and so the neuromorphic system can handle more complex tasks. I would like to know why the authors do not challenge such tasks. If the parity problem is the most difficult nonlinear task that can be performed with spiking neural networks, how are the studies on computational performance of spiking neural networks developed to the level useful for understanding human brain capable of more complex tasks?

The authors claim that the scale of the neural networks used for the emulation is small because of the limitation of the neuromorphic system, BrainScaleS 2. I don't know whether the emulation with this small-scale neuromorphic system is really significant or not.

Reviewer #3 (Remarks to the Author):

In this paper, the Authors investigated the relation between criticality and task-performance. For some small neural network (up to 32), they performed simulations on hardware neuromorphic chip (Brainscale2). They performed several simulations showing that simple tasks are suffering from criticality. Tuning the input force, the system can be closed to criticality or far depending the complexity of the studied tasks. This paper is well written and clear to read.
The authors answered to most of my comments and improved the paper adding two additional tasks and several detailed explanations including the limitations of the hardware neuromorphic board.

Minor revisions:
In Introduction part, the physical implementation part (line 78-79) referred to old work. I recommend to add also some recent works as:
S. Furber, D. Lester, L. Plana, J. Garside, E. Painkras, S. Temple, A. Brown, Overview of the SpiNNaker system architecture, IEEE Transactions on Computers, 62:12, 2454-2467, 2012
P. Merolla et al., A million spiking-neuron integrated circuit with a scalable communication network and interface, Science, 345:6197, August 2014
F. Khoyratee, F. Grassia, S. Saighi, T. Levi, Optimized real-time biomimetic neural network on FPGA for bio-hybridization, Frontiers in Neuroscience, 13-377, 2019
G. Indiveri et al., Neuromorphic silicon neuron circuits, Frontiers in Neuroscience, 5:73, 2011

Reviewer #2

The authors almost appropriately revised the manuscript according to the comments from the reviewers. In particular, the additional results on the computational performance under temporally switched inputs are interesting.

The two newly added tasks are not so complex compared with other practical temporal tasks. Any non-binary information can be in principle coded by binary information at a certain precision, and so the neuromorphic system can handle more complex tasks. I would like to know why the authors do not challenge such tasks. If the parity problem is the most difficult nonlinear task that can be performed with spiking neural networks, how are the studies on computational performance of spiking neural networks developed to the level useful for understanding human brain capable of more complex tasks?

There must be a misunderstanding. If the network is able to perform on the XOR or the *n*-bit parity task (and sum, *n*-sum, OR), then it can in principle do any other task. However, computational capacity in our case is limited, because the prototype chip is still quite small. For completeness, we now show results for the NARMA task as well (Fig. 5e). As expected the network shows fair performance on the NARMA task, given its size. Moreover, as shown previously (Boedecker et al, and Bertschinger & Natschläger 2004), the performance on the more complex NARMA task is better closer to criticality.

The authors claim that the scale of the neural networks used for the emulation is small because of the limitation of the neuromorphic system, BrainScaleS 2. I don't know whether the emulation with this small-scale neuromorphic system is really significant or not.

First, we want to clarify that the used neuromorphic chip is a prototype of the BrainScaleS 2 system. A full size version with 512 LIF neurons will be available in the future. Our work represents a stepping stone to larger systems. With our work, we demonstrate the principles of how to use the analog neuromorphic systems – which are prone to noise (thermal as well as fixed-pattern) – and how they can be used for actual computation. These principles are applicable to networks of any size, and are therefore of general relevance.

Reviwer #3

In this paper, the Authors investigated the relation between criticality and task-performance. For some small neural network (up to 32), they performed simulations on hardware neuromorphic chip (Brainscale2). They performed several simulations showing that simple tasks are suffering from criticality. Tuning the input force, the system can be closed to criticality or far depending the complexity of the studied tasks. This paper is well written and clear to read. The authors answered to most of my comments and improved the paper adding two additional tasks and several detailed explanations including the limitations of the hardware neuromorphic board.

Minor revisions: In the Introduction part, the physical implementation part (line 78-79) referred to old work. I recommend to add also some recent work.

We would really like to thank the reviewer for the encouraging feedback. As suggested, we included the references named by the reviewer in line 79 in the main manuscript. In addition, we added further citations to cover a broad range of neurormophic devices that are currently being developed.

REVIEWERS' COMMENTS:

Reviewer #2 (Remarks to the Author):

The authors addressed the previous comments and appropriately showed the results on additional experiments on the NARMA task.
The revised manuscript can be accepted.

Reviewer #3 (Remarks to the Author):

The authors improved their manuscript and answered to my comments.

Reviewer #2

The authors addressed the previous comments and appropriately showed the results on additional experiments on the NARMA task. The revised manuscript can be accepted.

We would really like to thank the reviewer for the encouraging feedback and the insightful comments.

Reviwer #3

The authors improved their manuscript and answered to my comments.

We would really like to thank the reviewer for the suggestions and the insightful comments.
